# Beginning the quest: phylogenetic hypothesis and identification of evolutionary lineages in bats of the genus *Micronycteris* (Chiroptera, Phyllostomidae)

**DOI:** 10.3897/zookeys.1028.60955

**Published:** 2021-04-06

**Authors:** Darwin M. Morales-Martínez, Hugo F. López-Arévalo, Mario Vargas-Ramírez

**Affiliations:** 1 Grupo de Biodiversidad y Conservación Genética, Instituto de Genética, Universidad Nacional de Colombia, Universidad Nacional de Colombia, Carrera 45 No 26-85, Bogotá, Colombia Universidad Nacional de Colombia Bogotá Colombia; 2 Grupo en Conservación y Manejo de Vida Silvestre, Instituto de Ciencias Naturales, Universidad Nacional de Colombia, Carrera 45 No 26-85, Bogotá, Colombia Universidad Nacional de Colombia Bogotá Colombia; 3 Estación de Biología Tropical Roberto Franco, Universidad Nacional de Colombia. Carrera 33 #33-76, Barrio El Porvenir, Villavicencio, Meta, Colombia Universidad Nacional de Colombia Bogotá Colombia

**Keywords:** Distribution, neotropical bats, species delimitation, systematics, taxonomy

## Abstract

Thirteen species of Neotropical bats of the genus *Micronycteris* are currently recognized and are allocated to four subgenera *Leuconycteris*, *Micronycteris*, *Schizonycteris*, and *Xenonectes*. Despite that, the presence of polyphyletic clades in molecular phylogenies suggests that its diversity is underestimated. Additionally, the incorrect identification of some genetic sequences, the incorrect assignation of available valid names, and restricted geographic sampling have biased the identification of independently evolutionary lineages within *Micronycteris*. In this study, several unknown genealogical lineages in the genus are identified and an updated phylogenetic hypothesis is proposed using mitochondrial and nuclear DNA fragments. The phylogenetic analyses congruently showed all individuals in four well-supported subgenera, but *M. schmidtorum* was revealed as the sister taxon of *M. brosseti* in the subgenus Leuconycteris. Twenty-seven different genealogical lineages were identified. These included eight confirmed species: *M. brosseti*, *M. buriri*, *M. giovanniae*, *M. matses*, *M. schmidtorum*, *M. simmonsae*, *M. tresamici*, and *M. yatesi*. Nineteen either allopatric or parapatric candidate species were also confirmed, two within the *M. hirsuta* complex, nine within the *M. megalotis* complex, seven within the *M. minuta* complex, and one corresponding to “*M.* sp.”. These results revealed an extensive undescribed diversity within each subgenus of *Micronycteris*. Nevertheless, the evolutionary processes associated with the specific radiations are poorly understood. This is just the beginning of the assessment of the taxonomy and systematics of *Micronycteris*, which requires additional integrative taxonomical approaches for its advance.

## Introduction

Scientists describe between 200 and 300 mammal species per decade, mainly small species like rodents and bats (Patterson 2000; Reeder et al. 2007). Recent increases in species descriptions are due to discoveries based on fieldwork (Reeder et al. 2007), and the application of the genetic and phylogenetic species concepts (Solari and Martínez-Arias 2014). This is especially true for Neotropics, where a larger number of undiscovered mammal species remain undescribed (Patterson 2000; Reeder et al. 2007).

In particular, bats (Chiroptera) represent a highly diverse mammal group in the Neotropics, comprising 21% (1386 species) of the mammal diversity (6495 species) and with an elevated number of species described in the last 10 years (Burgin et al. 2018). Among the vast diversity of neotropical bats, phyllostomids represent the largest recent radiation with ca. 223 species currently recognized (Simmons and Cirranello 2020) and 59 species described or split since 2005 (Burgin et al. 2018).

Within Phyllostomidae, bats of the genus *Micronycteris* are gleaning insectivores that are common in Neotropical bat assemblages. This genus currently comprises 13 recognized species allocated to four subgenera (i.e., *Leuconycteris*, *Micronycteris*, *Schizonycteris*, and *Xenonectes*; Porter et al. 2007), and several studies using genetic data have suggested that the diversity of this group has been underestimated (Porter et al. 2007; Larsen et al. 2011; Siles et al. 2013; Siles and Baker 2020). At least, two widely distributed species, *M. megalotis* and *M. minuta* may have possible cryptic lineages because phylogenetic assessments for both species recovered strongly supported polyphyletic clades (Porter et al. 2007). Furthermore, some inconsistencies in the identity of the species assigned to the different clades have been identified: (1) some GenBank sequences lack voucher specimens and/or the confirmation of the sequence identification (e.g., Porter et al. 2007), (2) some names have been changed among phylogenetic hypotheses without robust arguments (e.g., Porter et al. 2007; Larsen et al. 2011; Siles et al. 2013), (3) some clades names were based on sequences from specimens collected far from the type locality without justification (e.g., *M. microtis* in Siles et al. 2013) and (4) the geographic coverage of the sequences has been extremely limited, with sequences from the central distribution of the genus from countries with a high diversity of the genus such as Colombia being practically nonexistent (see. Porter et al. 2007). Therefore, the correct interpretation of the diversity within *Micronycteris* depends on analyses including the assessment of genetic variation within the genus, incorporating vouchers from poorly surveyed portions of its distribution range.

Despite the evidence of cryptic diversity within *Micronycteris* (Porter et al. 2007; Larsen et al. 2011; Siles et al. 2013; Siles and Baker 2020), the identification of cryptic lineages has lacked a logical framework for their accurate delimitation. Such accurate lineage delimitation is critical for the assessment of priority areas for conservation (Vieites et al. 2009; Burgin et al. 2018), the monitoring of biodiversity loss (Vieites et al. 2009), the identification of potential vectors of zoonotic diseases (Bickford et al. 2007), the evaluation of biological interactions, and the formulation and assessment of evolutionary or biogeographic hypotheses (Burgin et al. 2018).

In this study we aimed at assessing the diversity and evolutionary relationships of the lineages making up the genus *Micronycteris* by identifying a yet-undescribed portion of its genetic diversity and at proposing an updated phylogenetic hypothesis. For this, we analyzed a combination of molecular data including fragments of the cytochrome-b gene (Cytb) mitochondrial DNA (mtDNA) and the intron 7 of the nuclear ﬁbrinogen, B beta polypeptide gene (Fgb-I7) nuclear DNA (nDNA). Furthermore, we included new sequences from individuals of several species of *Micronycteris* from wider geographical distribution.

## Methods

### Revision of specimens

To confirm the correct identification of the sequences in our analyses, we examined at least one voucher specimen from most of the clades reported in previous studies (Porter et al. 2007; Larsen et al. 2011; Siles et al. 2013; Siles and Baker 2020). The examined specimens (Suppl. material 1: Table S1) are housed in the following collections: The American Museum of Natural History (**AMNH**), USA; the Instituto de Ciencias Naturales of Universidad Nacional de Colombia (**ICN**), Colombia and the Pontificia Universidad Católica del Ecuador (**PUCE**), Ecuador.

### Laboratory procedures

We extracted genomic DNA using the phenol-chloroform method (Sambrook et al. 1989) from fresh samples of liver or muscle tissues preserved in ethanol, and for museum specimens, we extracted punches of tissue from the plagiopatagium. We obtained DNA from individuals of five species, *M. hirsuta*, *M. megalotis*, *M. microtis*, *M. minuta*, and *M. schmidtorum* from several localities in Colombia (Suppl. material 2: Fig. S1). We amplified 32 sequences between 700 and 1100 base pairs (bp) of the cytochrome-b gene (Cytb) mitochondrial DNA (mtDNA) and 19 sequences between 500 and 700 bp of the fibrinogen beta chain (Fgb-I7) gene nuclear DNA (nDNA) from the newly obtained specimens. We used the same primers and followed the same laboratory protocols and methods of Porter et al. (2007) for both genes, albeit increasing the annealing temperature from 45–48 °C to 50.5 °C for the Cytb amplification. Additionally, we gathered 101 sequences of Cytb and Fgb-I7 of *Micronycteris* species from the GenBank and Bold Systems databases and 64 sequences provided in Siles and Baker (2020) and included in the analyses (Suppl. material 1: Table S1).

### Phylogenetic analyses

For the phylogenetic analyses, we included 198 sequences of Cytb (mtDNA) comprising all currently recognizes species of *Micronycteris*, 150 sequences of Fgb-I7 (nDNA), and 146 individuals with complete data set (Cytb + Fgb-I7) of most of the *Micronycteris* species except by *M. sanborni* (list of specimens and sequence numbers in Suppl. material 1: Table S1), covering a wide portion of the geographic distribution of the genus (see Suppl. material 1: Table S1). We performed multiple sequence alignment for both genes using the Clustal W algorithm in BioEdit 7.2.6 software (Hall 1999). We analyzed the CytbmtDNA, the Fgb-I7nDNA, and the complete evidence (Cytb + Fgb-I7) data sets, using the following partition scheme: (i) unpartitioned, (ii) partitioned by gene (i.e., each gene fragment treated as a distinct partition) and (iii) maximum partitioning (i.e., each codon of the protein-coding gene Cytb and the Fgb-I7 gene fragment treated as distinct partitions). We assessed the optimal partitioning scheme and best-fit evolutionary models using PartitionFinder v2 and the Bayesian Information Criterion (Lanfear et al. 2016) and selected the maximum partitioning scheme. We applied the following resulting models in a Bayesian analysis (BA) with MrBayes v 3.2.1 (Ronquist et al. 2012): Cytb 1^st^ codon – K80+G, Cytb 2^nd^ codon – K81uf+I+G, Cytb 3^rd^ codon – K80+G and Fgb-I7– HKY+I+G. Those models were incorporated into a single tree search (mixed model partition approach; Nylander et al. 2004), and two parallel runs were carried out using four Markov chains, run for 50 million generations, sampling every 100 generations. We discarded 25% of the resulting trees as burn-in, and 85% of the trees were used for generating a 50% majority-rule consensus. We assessed an acceptable level of the MCMC chains and estimated the effective sample sizes for all parameters using the software Tracer 1.5.4 (Rambaut et al. 2018). Additionally, we performed phylogenetic analyses of the same data sets using the Maximum Likelihood algorithm implemented in RAxML 1.5 beta software (Stamatakis 2014). The default GTR+G model was set across all partitions. Five independent Maximum Likelihood searches were performed with different starting conditions and the rapid bootstrap algorithm to explore the robustness of the branching patterns by comparing the best trees. Afterwards, 1000 non-parametric thorough bootstrap values were computed and plotted against the best tree. For all phylogenetic analyses, we used homologous sequences of other bat species including *Desmodus rotundus*, *Glossophaga soricina*, *Hsunycteris cadenai*, *Hsunycteris thomasi*, *Lampronycteris brachyotis*, *Lionycteris spurrelli*, and *Lonchophylla robusta* as outgroups (Suppl. material 1: Table S1). We based the description of the resulting phylogenetic hypothesis for the genus *Micronycteris* on the complete evidence data set analyses.

### Lineage delimitation analyses

We evaluated whether populations within the genus *Micronycteris* corresponded to different independently evolving evolutionary lineages (the General Lineage Species Concept of de Queiroz 1999; 2007) when two or more independent lines of evidence supported their distinctiveness. For that, we used the following different lines of evidence: (1) identification of monophyletic lineages in the mtDNA and mtDNA + nDNA phylogenies, (2) assessment of genetic distances using the mitochondrial gene, (3) identification of mitochondrial monophyletic clades matching unique nDNA haplotypes, and (4) the use of single locus species delimitation methods. Based on the concordance of those lines of evidence we identified whether those lineages corresponded to Confirmed Species or Confirmed Candidate Species (CCS); the later sensu Vieites et al. (2009) defined as: generally deep Cytb lineages (> 3% sequence divergence), differing clearly by morphology or with exclusively nDNA private haplotypes and recognized by at least one species delimitation method.

We started the lineage delimitation by determining the monophyletic clades through the phylogenetic analyses and afterward we searched for mitochondrial lineages with genetic divergences > 3%, using the Species Identifier ‘Cluster’ algorithm in Taxon DNA 1.7 (Meier et al. 2006). We used 3% because it was the percentage of the degree of genetic divergence that often corresponds to species-level units in several phyllostomid bats (see Genetic Species Concept in Bradley and Baker 2001 and Baker and Bradley 2006). We assessed the genetic distances between clades using uncorrected *p*-distances, implemented in the software Mega v.10.0.5 (Kumar et al. 2018).

Then, we searched for genealogical concordance between mitochondrial and nuclear lineages because such concordance has been considered an essential criterion for species recognition (Avise and Ball 1990). For that, we searched for unique haplotypes in the Fgb-I7 gene corresponding to the mtDNA lineages revealed by the phylogenetic trees and showing genetic distances of > 3%. We trimmed all sequences of Fgb-I7 to equal length and removed the sequences containing ambiguities that could not be interpreted as heterozygotes. We resolved nuclear DNA sequences to haplotypes with the PHASE algorithm (Stephens et al. 2001) implemented in the software DNASp 6 (Rozas et al. 2017). The phased sequences were then used to construct a haplotype network in the software Haplotype Viewer (http://www.cibiv.at/~greg/haploviewer), based on a Neighbour-joining tree from uncorrected *p*-distances computed with MEGA v.10.0.5 (Kumar et al. 2018).

In addition, we performed the following three different single-locus species delimitations models: (i) The Bayesian implementation of Poisson tree processes (bPTP), (ii) The single-threshold method of the generalized mixed Yule coalescent model (GMYC), and (iii) The multi-rate Poisson tree processes for single locus (mPTP). All analyses were performed using the Exelixis Lab’s web server (bPTP – http://species.h-its.org/ptp/; mPTP – https://mptp.h-its.org/#/tree; GMYC – http://species.h-its.org/gmyc/). For the delimitation analyses, we used unique haplotypes of Cytb across the aligned region to avoid the influence of duplicate haplotypes in the analyses (147 terminals; Suppl. material 1: Table S1). We constructed an ultrametric tree with BEAST v1.7.2 (Drummond et al. 2012) using a lognormal relaxed-clock model, a coalescent constant-size tree prior, and a relative time set with a prior on the ingroup age of one (normal distribution: mean = 1, SD = 0.01). We ran two independent MCMC analyses for 50 million generations, sampling every 1000 generations. We assessed convergence, discarded a fraction of trees as burn-in, and summarized the trees as in MrBayes phylogenetic analyses above. The resulting tree was used for the delimitation analyses. The bPTP delimitation search was performed for 500,000 Markov chain Monte Carlo (MCMC) generations, with thinning set to 100 and a burn-in of 25% of initial samples. The convergence of bPTP analysis was visually checked in the trace plot. Finally, we considered both, the Maximum likelihood and Bayesian solutions for the bPTP delimitation.

### Data resources

The data underpinning the analysis reported in this paper are deposited in the Mendeley Repository at: http://dx.doi.org/10.17632/vyp75f243x.1

## Results

### Phylogenetic analyses

**Figure 1.**
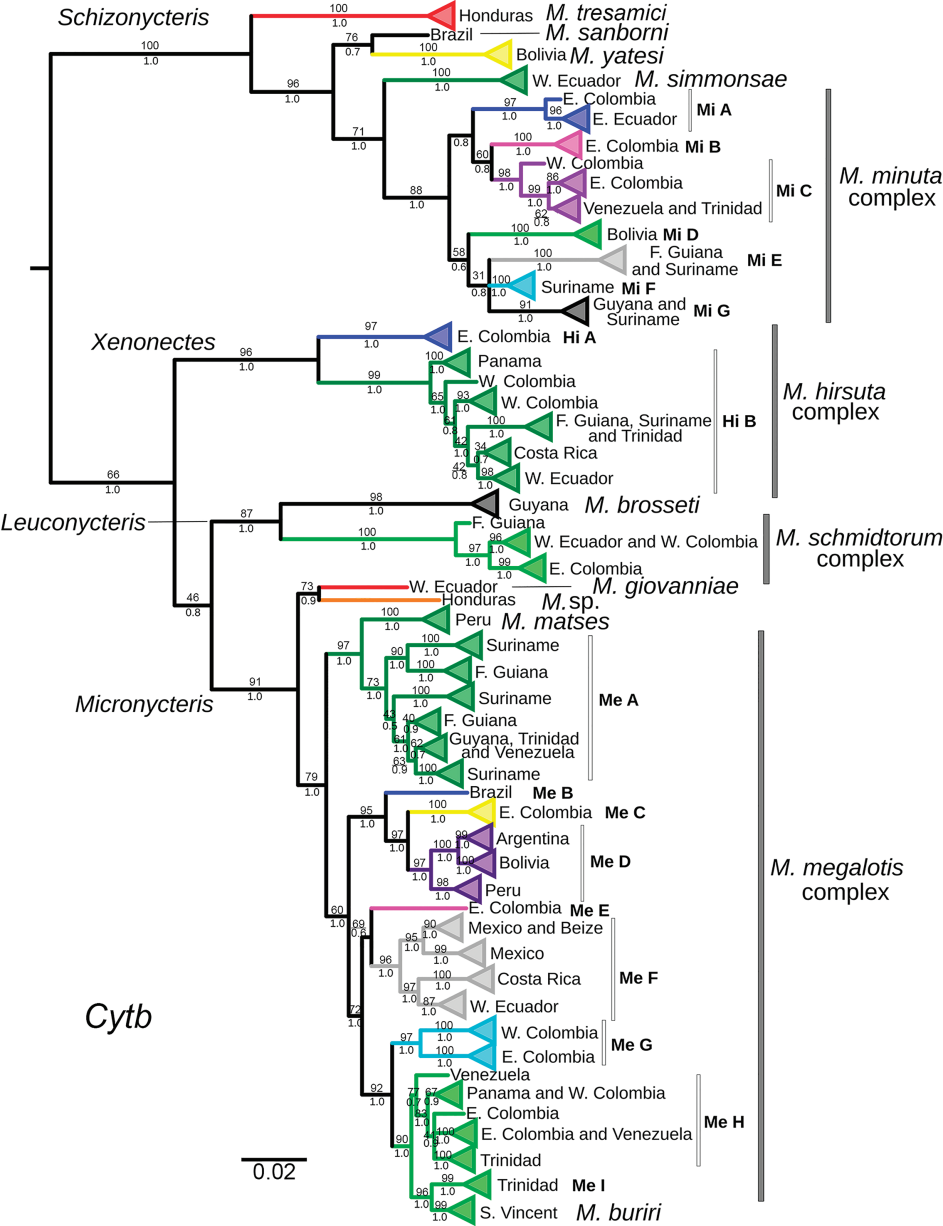
Bayesian phylogram of the genus *Micronycteris* from the phylogenetic analysis of the Cytb gene, mtDNA. Numbers below the nodes correspond to Bayesian posterior probabilities, and those above correspond to bootstrap support values (percentages). Colors indicate the clades with > 3% of genetic differentiation. Sequences within groups are listed in Suppl. material 1: Table S1 and depicted in Fig. 5. Bootstrap support values are missing in clades not recovered by ML analyses. *Micronycteris sanborni* was not included in mitochondrial genetic differentiation analyses because it is a chimeric sequence of two individuals.

**Figure 2.**
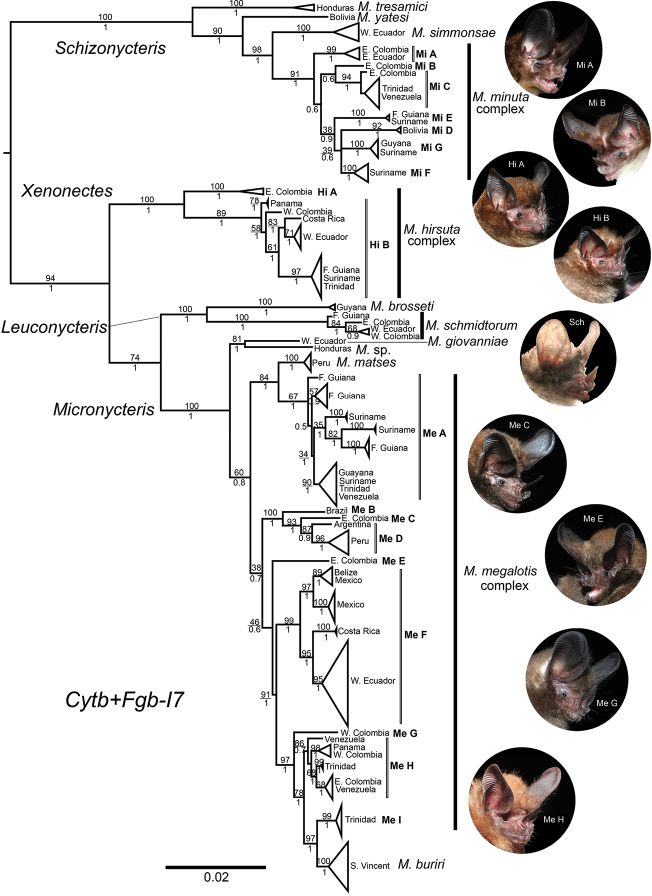
Bayesian phylogram of the genus *Micronycteris* from the phylogenetic analysis of combined evidence (mtDNA + nDNA). Numbers below the nodes correspond to Bayesian posterior probabilities, and those above correspond to bootstrap support values (percentages). The acronyms Mi, Hi, and Me represent the candidate species for *M. minuta*, *M. hirsuta* and *M. megalotis*, respectively. Sequences per group are listed in Table S1. Inset photos: (Mi A) ICN-24465♀: Colombia, Caquetá, Florencia, Vereda el Venado, Macagual; (Mi B) ICN 23912♂: Colombia, Vichada, Cumaribo, Matavén River; (Hi A) ICN-23867♀: Colombia, Guaviare, San José del Guaviare, Vereda Los Alpes, El Provenir farm; (Hi B) ICN Temporal D3M541♂, Colombia, Santander, Puerto Parra, Bocas del Carare; (Sch) ICN-24479♂: Colombia, Magdalena, Santa Marta, Tayrona National Natural Park; (Me C) ICN-23869♂: Colombia, Guaviare, San José del Guaviare, Vereda Los Alpes, El Provenir farm; (Me E) ICN-23839♂: Colombia, Guaviare, San José del Guaviare, Vereda El Raudal, Angosturas II; (Me G) ICN-24495♀: Colombia, Huila, Gigante, Vereda Matambo, La Ensillada farm; (Me H) ICN-23203♂. Colombia, Meta, La Macarena, Vereda Caño Canoas, High plain of Caño Canoas.

For the Cytb gene dataset and the complete evidence, both tree building methods placed all individuals into four well-supported major clades that corresponded to four subgenera of *Micronycteris* namely: *Leuconycteris*, *Micronycteris*, *Schizonycteris*, and *Xenonectes*, (Fig. 1). However, the phylogenetic position of *M. schmidtorum* was placed in the subgenus Leuconycteris (Figs 1, 2). *Leuconycteris* included the species *M. brosseti* and *M. schmidtorum*. *Micronycteris* included the species *M. buriri*, *M. giovanniae*, *M. matses*, an undescribed species “*M.* sp.”, and nine clades within *M. megalotis* complex (including *M. microtis*, see taxonomic comments in discussion). *Schizonycteris* included the species *M. tresamici*, *M. sanborni*, *M. yatesi*, *M. simmonsae*, and seven clades within the *M. minuta* complex. Finally, *Xenonectes* contain two clades of *M. hirsuta* complex (Figs 1, 2).

The first strongly supported major clade matched the subgenus Schizonycteris (Fig. 2). Within this major clade, the first strongly supported subclade corresponded to the species *M. tresamici* from Honduras. This subclade was recovered basal to the strongly supported species *M. yatesi* (BA: 1, ML: 90%) from Bolivia. The latter was recovered with robust support (BA: 1, ML: 98%) basal to the species *M. simmonsae* from Ecuador. These subclades were recovered by both phylogenetic analyses as successive sister taxa of seven subclades comprising the *M. minuta* complex (denoted with Mi abbreviature; Fig. 2). This complex is represented by two reciprocally monophyletic genetic groups; the first is robustly supported (BA: 1, ML: 99%) and included individuals from eastern Ecuador and Colombia (Mi A), the second showed lower support (not recovered in the ML analyses) included two genetic subgroups. The first subgroup contained an individual from eastern Colombia (Mi B), and its sister robustly supported subgroup (BA: 1, ML: 94%), included individuals from Colombia, Trinidad, and Venezuela (Mi C). The second weakly supported genetic group (BA: 0.9, ML: 38%) included a first strongly supported subgroup (BA: 1, ML: 100%) including individuals from French Guiana and Suriname (Mi E), and a second low supported subgroup made up of three strongly supported subclades including individuals from Bolivia (Mi D; BA: 1, ML: 92%), Guyana and Suriname (Mi G; BA: 1, ML: 100%), and Suriname (Mi F; BA: 1, ML: 100%; Fig. 2).

The second strongly supported major clade, matching the subgenus Xenonectes, corresponded to the *M. hirsuta* complex. This major clade was formed by two strongly supported monophyletic subclades (denoted with Hi abbreviature; Fig. 2). The first corresponded to a strongly supported subclade from eastern Colombia (Hi A; Fig. 2). The second is a strongly supported subclade (BA: 1, ML: 89%) included individuals from Panama, western Colombia, Costa Rica, French Guiana, Suriname, Trinidad, and western Ecuador (Hi B; Fig. 2).

The third major clade corresponded to the subgenus Leuconycteris and included two strongly supported reciprocally monophyletic subclades. The first subclade constituted by *M. brosseti* from Guyana; and the second subclade by *M. schmidtorum*, including individuals from French Guiana, western Ecuador, and both western and eastern Colombia (Fig. 2).

Finally, the fourth major clade represented by the subgenus Micronycteris included three inclusive reciprocally monophyletic subclades (Fig. 2). The first robustly supported subclade (BA: 1, ML: 81%) comprised the species *M. giovanniae*, formed by an individual from western Ecuador, and as the sister taxon to an undescribed species “*M.* sp.”., from Honduras (Fig. 2). The second moderately supported monophyletic subclade (BA: 1, ML: 84%), comprised a strongly supported lineage corresponding to the species *M. matses*. This lineage represented by individuals from eastern Peru, and appeared as sister of a weakly supported genetic group (BA: 1, ML: 67%) composed of individuals from French Guyana, Surinam, Guyana, Trinidad, and southern Venezuela (Me A; Fig. 2). Finally, the third weakly supported monophyletic subclade (BA: 0.7, ML: 38%) comprised two genetic groups. The first group was a robustly supported lineage formed by an individual from Brazil (Me B), as the sister taxon of a robustly supported genetic group (BA: 1, ML: 93%) formed by an individual from eastern Colombia (Me C) and a robustly supported lineage (BA: 0.9, ML: 87%) composed by individuals from Argentina and Peru (Me D; Fig. 2). The second genetic group was recovered with weak support (BA: 0.6, ML: 46%), including an individual from eastern Colombia (Me E), as the sister of a lineage composed by two genetic subgroups. The first one corresponded to a strongly supported subgroup (BA: 1, ML: 99%), comprising individuals from Belize, Mexico, Costa Rica, and western Ecuador (Me F; Fig. 2). This genetic subgroup was shown to be the sister of a robustly supported subgroup (BA: 1, ML: 97%) formed by an individual from western Colombia (Me G), as the sister of a robustly supported genetic subgroup comprising a medium supported lineage (BA: 0.7, ML: 86%) including individuals from western Colombia, Panama, eastern Colombia, southern Venezuela (Me H; Fig. 2). The later genetic subgroup was revealed as the sister of a strongly supported subgroup that comprised a supported lineage (BA: 1, ML: 99%) from Trinidad and Tobago (Me I; Fig. 2), being sister of a strongly supported clade of *M. buriri* form Saint Vincent Island.

### Lineage delimitation

The “Cluster” algorithm of TaxonDNA revealed 24 reciprocally monophyletic clades with genetic divergence of more than 3% (Fig. 1, colored clades). Within the subgenus Schizonycteris we found ten lineages, representing to *M. yatesi*, *M. tresamici*, *M. simmonsae*, and seven clades belonging to the *M. minuta* complex (Fig. 1; Suppl. material 3: Table S2). Within the subgenus Xenonectes, we found two subclades with > 3% of genetic distance (Fig. 1; Suppl. material 3: Table S2). Within the subgenus Leuconycteris, we found two subclades with > 3% of genetic distance, one corresponding to the recognized species *M. brosseti*, and the second by the species *M. schmidtorum* (Fig. 1; Suppl. material 3: Table S2). Lastly, within the subgenus Micronycteris we found ten subclades with > 3% genetic distance between them, including (i) *M. giovanniae*, (ii) an undescribed species (“*M.* sp.”), and (iii) eight clades forming the *M. megalotis* complex (Fig. 1; Suppl. material 3: Table S2). The nominal species *M. matses* and *M. buriri* failed to have a > 3% of the genetic distance with any other subclade (Suppl. material 3: Table S2; Fig. 1).

**Figure 3.**
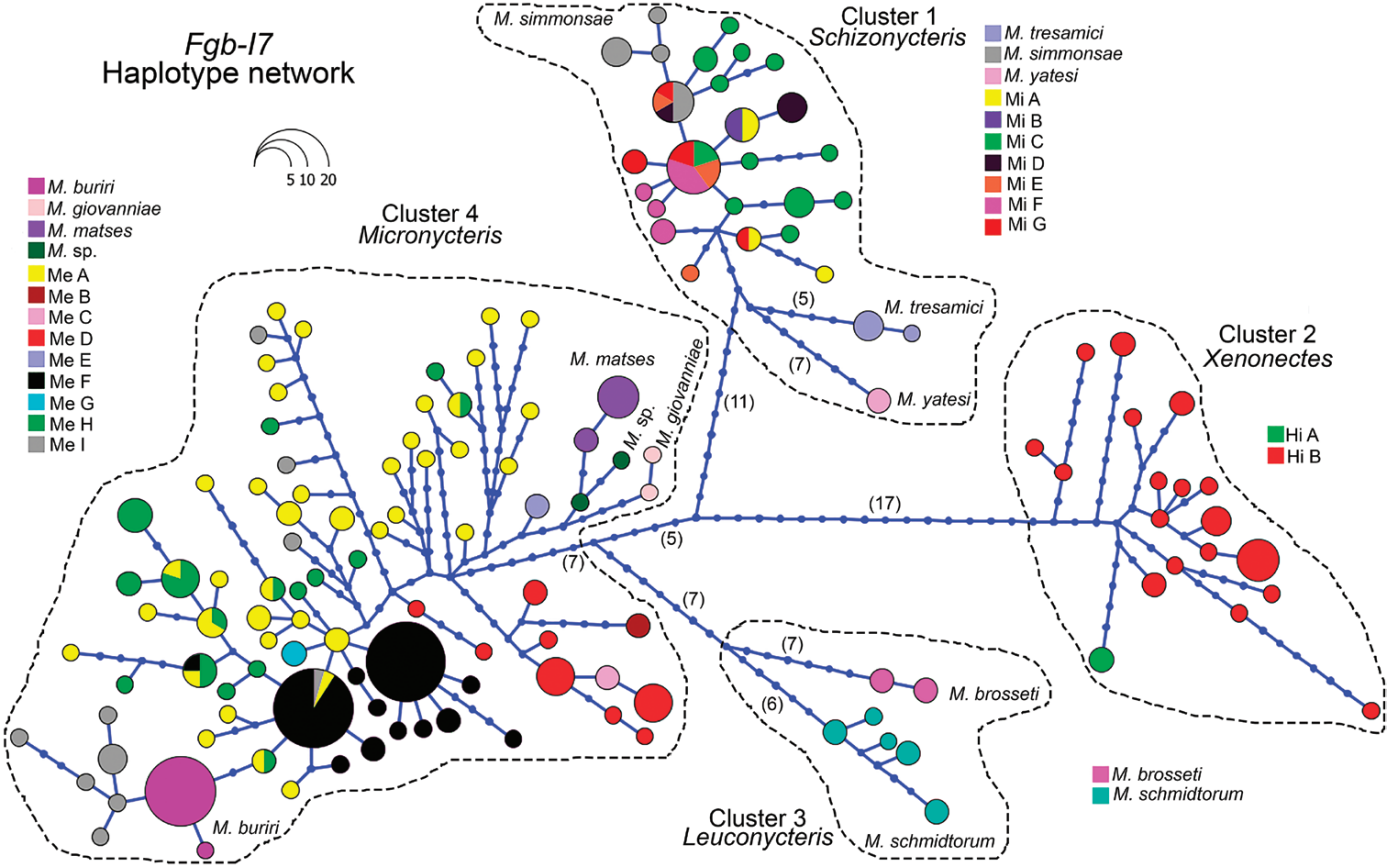
Haplotype network of the genus *Micronycteris* for the Fgb-I7 gene nDNA haplotypes based on an alignment of 686 bp. Circle size reflects haplotype frequency and missing haplotypes are represented by small circles. Each line connecting haplotypes corresponds to one mutational step. Colors within each nominal species haplogroup represent the candidate species and Mi, Hi, and Me correspond to their acronyms in *M. minuta*, *M. hirsuta*, and *M. megalotis* respectively.

**Figure 4.**
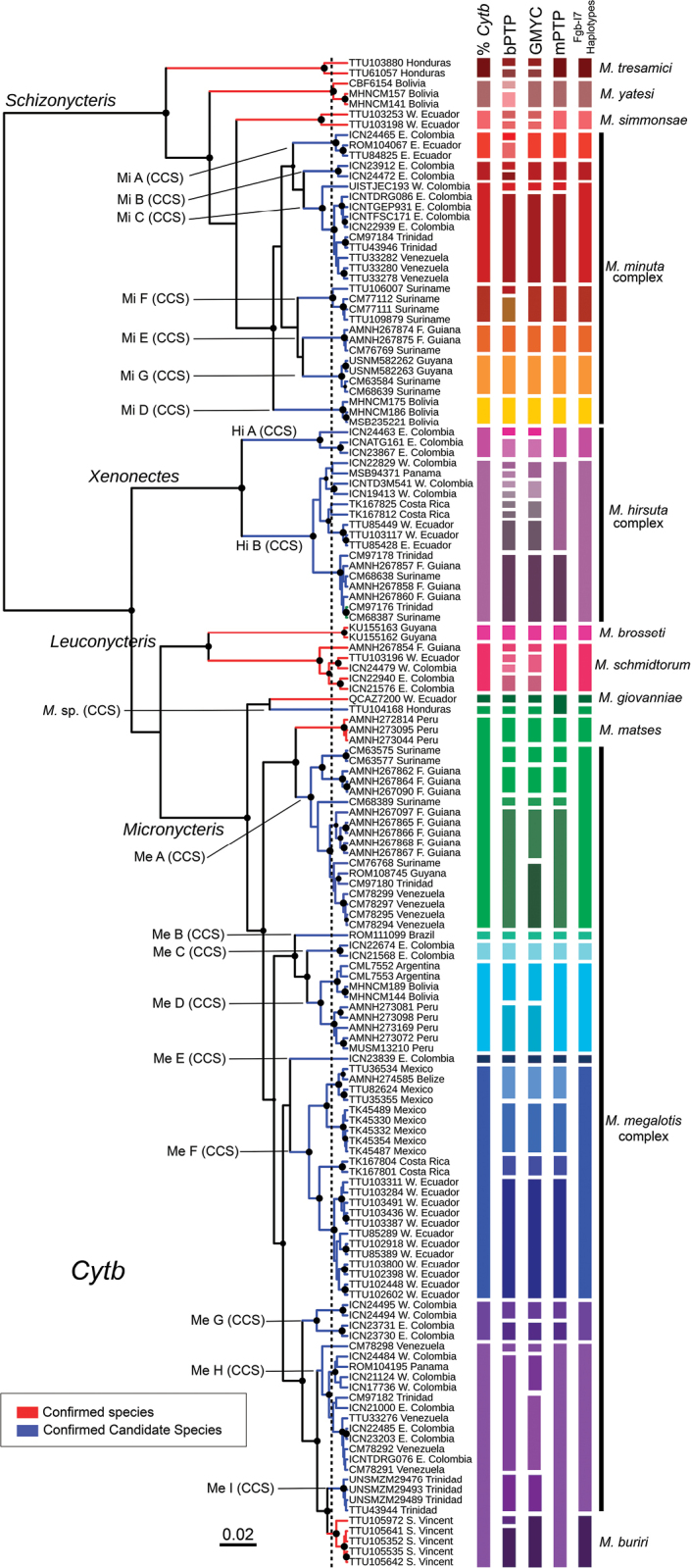
Bayesian phylogram of the genus *Micronycteris* from the analysis of Cytb gene mtDNA (BEAST), showing colored bars that represent the different delimitation schemes obtained with > 3% of genetic differentiation, bPTP, GMYC, mPTP, and Fgb-I7 haplotype network. CS: Confirmed species (Red), CCS: Confirmed Candidate Species (Blue). The dashed vertical line indicates the threshold between the Yule and the coalescent process estimated by the likelihood implementation of the GMYC model. Filled circles at internal nodes indicated strong support for Bayesian (BEAST: PP > 0.95), and the size is proportional between 0.95 to 1 PP. The acronyms Mi, Hi, and Me represent the candidate species in *M. minuta*, *M. hirsuta*, and *M. megalotis*, respectively. Sequences within groups are listed in Suppl. material 1: Table S1 and depicted in Fig. 5.

Our Fgb-I7 gene TCS haplotype network contained four genetic clusters separated by a minimum of 23 mutational steps (Fig. 3). These clusters corresponded to 130 haplotypes that matched the 24 mitochondrial lineages identified by the TaxonDNA analyses (> 3% genetically of divergence), and *M. matses* and *M. buriri* (that have < 3% of genetic distance; Fig. 1), all recovered by the complete evidence phylogeny too (Figs 2, 4). The subgenus Schizonycteris (Cluster 1) was formed by 27 haplotypes, matching the three recognized species (*M. tresamici*, *M. yatesi*, and *M. simmonsae*) and the seven mitochondrial lineages within the *M. minuta* complex (Mi A–Mi G) separated between one and 12 mutational steps. The subgenus Xenonectes (*M. hirsuta* complex. Cluster 2) was formed by 19 haplotypes that corresponded to the two mtDNA lineages Hi A and Hi B, separated by a minimum of seven mutational steps. The subgenus Leuconycteris (Cluster 3) included *M. schmidtorum* with five haplotypes and *M. brosseti* with two haplotypes, separated by a minimum of 13 mutational steps from the haplotypes of *M. schmidtorum* (Fig. 3). Finally, the most extensive haplotype cluster matched the subgenus Micronycteris (Cluster 4), formed by haplotypes that diverged between one to 27 mutational steps from each other. Unique haplotypes in this haplogroup matched three species (i.e., *M. matses*, *M. giovanniae*, and “*M.* sp.”) and nine lineages forming the *M. megalotis* complex (Fig. 3).

The single locus species delimitation analyses revealed contrasting results. The bPTP model delimited 55 entities. Nevertheless, only 15 of those clades showed posterior probabilities above 0.95 (Suppl. material 4). The other clades showed between 0.49 and 0.95, which suggests that caution should be considered when interpreting these clades as independently evolving lineages (Suppl. material 4). The single-threshold method of the GMYC model delimited 48 entities. The model was significantly better than the null hypothesis with the likelihood ratio test (LR = 5040.823, LRT results = 0***, threshold time = -0.008590693; Suppl. material 4). Finally, the mPTP model was somewhat more conservative, identifying 33 species (Fig. 4) that matched several lineages delimited by the bPTP and GMYC methods (Suppl. material 4).

**Figure 5.**
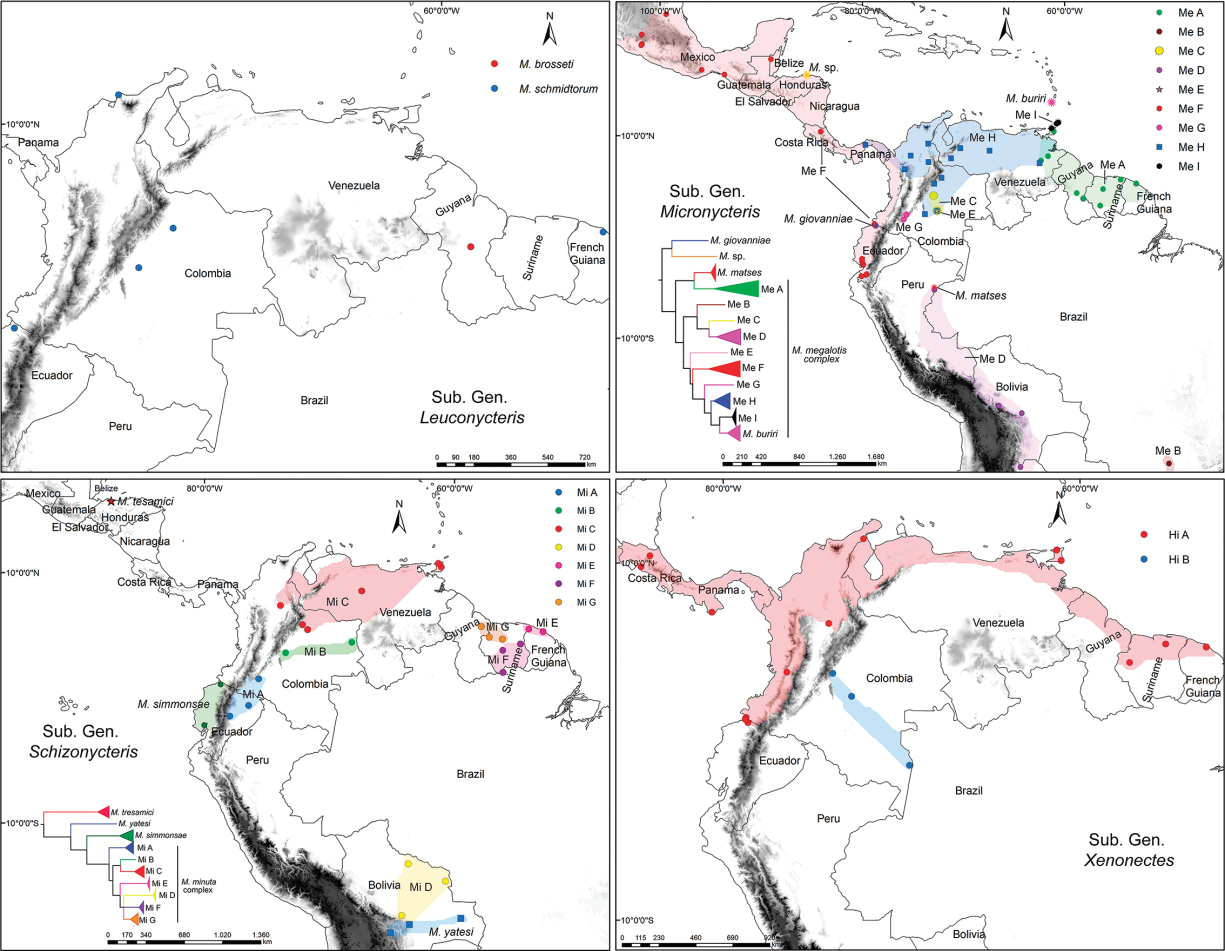
Geographic location of the individuals from which the sequences were obtained and included in the phylogenetic analyses. Shades represent a hypothetic estimation of the distribution of each clade based on the locality of the included individuals. The acronyms Mi, Hi, and Me represent the candidate species in *M. minuta*, *M. hirsuta*, and *M. megalotis*, respectively. Sequences within groups are listed in Suppl. material 1: Table S1.

Considering the different lines of evidence, we revealed 27 different genealogical lineages forming the genus (Fig. 4). They belong to eight confirmed species (CS): *M. brosseti*, *M. buriri*, *M. giovanniae*, *M. matses*, *M. schmidtorum*, *M. simmonsae*, *M. tresamici*, and *M. yatesi*, and 19 confirmed candidate species (CCS) with either allopatric or parapatric distribution within three species complexes. We present in Fig. 4 the position of each revealed evolutionary lineage in the Cytb tree and in Fig. 5 their hypothesized geographic distribution.

### Confirmed candidate species (CCS)

We found seven CCS within the sub genus *Schizonycteris*, in the *M. minuta* complex: (1) lineage Mi A formed by individuals from the Amazon of Ecuador and Colombia; (2) lineage Mi B formed by individuals from the north Amazon of Colombia; (3) lineage Mi C formed by individuals from dry-forest of western Colombia, the Orinoco Llanos of Colombia and Venezuela, and the Trinidad Island; (4) lineage Mi D formed by individuals from Bolivia; (5) lineage Mi E formed by individuals from north French Guiana and Suriname; (6) lineage Mi F formed by individuals from south Suriname and (7) lineage Mi G formed by individuals from north Guyana and Suriname. Two more CCS were confirmed within the subgenus Xenonectes in the *M. hirsuta* group: (1) lineage Hi A, including individuals from eastern Colombia and (2) lineage Hi B, including individuals from Costa Rica, Trinidad, French Guyana, Panama, Suriname, western Colombia, and western Ecuador. Finally, ten CCS within the subgenus Micronycteris: (1) lineage “*M.* sp.” from Honduras; (2) lineage Me A including individuals from Suriname, French Guyana, easternmost Venezuela, Guyana, and Trinidad; (3) lineage Me B including an individual from Brazil (this lineage could represent the nominal *M. megalotis* due to its type locality “unknown locality in Brazil”); (4) lineage Me C including individuals from north Amazon of Colombia; (5) lineage Me D comprising individuals from Argentina, Bolivia, and Peru; (6) lineage Me E including an individual from north Amazon of Colombia; (7) lineage Me F including individuals from Belize, Costa Rica, Mexico, and western Ecuador; (8) lineage Me G including individuals from the Magdalena valley and the eastern versant of the Andean Cordillera of Colombia; (9) lineage Me H formed by individuals from Panama, the dry forest of western Colombia and the Orinoco Llanos of Colombia and Venezuela and (10) lineage Me I formed by individuals from Trinidad. Due to the distribution of the Me F and Me H, two available names, *Micronycteris mexicana* and *Micronycteris microtis* might be applied to these lineages. Nonetheless, the applications of these names depend on a posterior taxonomic revision. We make no conclusions about the status of *M. sanborni* because we did not have enough information to include it in the applied framework.

## Discussion

Our results showed contrasting evolutionary patterns between the different subgenera of *Micronycteris*. At the specific level, the evolutionary histories of widely distributed species such as *M. megalotis*, *M. hirsuta*, and *M. minuta* are more complex than previously known (Porter et al. 2007; Larsen et al. 2011; Siles et al. 2013), with several unknown genealogical lineages with allopatric and/or parapatric distributions. The different specific factors that may have influenced lineage differentiation within the genus *Micronycteris* are still unknown, however, distinctive ecological and ethological characteristics such as the size of familiar groups, home range, and movement behavior may have played a prominent role, because it is known that small social groups, rigid social structures, and low dispersal capacity induce high genetic variation (Bradley and Baker 2001). Generally, *Micronycteris* contains widely distributed species that use various types of refuges in small social groups (i.e., familiar groups with fewer than 12 individuals. Emmons 1997); and they tend to have high refuge fidelity (Kalka and Kalko 2006; Albrecht et al. 2007). In addition, due to its foraging strategy, the species of *Micronycteris* do not frequent open areas (Albrecht et al. 2007). Bats of the genus *Micronycteris* have small home ranges (ca. 3.8 ha; Albrecht et al. 2007) compared to other phyllostomid bats such as the insectivores *Macrophyllum macrophyllum* (24 ha ranging from 7–151 ha; Meyer et al. 2005), *Lophostoma silvicolum* (11–31 ha; Kalko et al. 1999), *Lampronycteris brachyotis* (22–27 ha; Weinbeer and Kalko 2004), *Trachops cirrhosus* (46 ha ranging from 8–100 ha; Kalko et al.1999). A similar situation occurs when compared with other frugivorous phyllostomid bats such as *Carollia perspicillata* (155 ha; Bernard and Fenton 2003), *Sturnira lilium* (36–90.7 ha; Loayza and Loiselle 2008), *Artibeus watsoni* (1.8–17.9 ha; Albrecht et al. 2007), and nectarivores bats like *Lonchophylla dekeyseri* (640 ha ranging from 230–1453 ha; Aguiar et al. 2014), and *Glossophaga soricina* (660 ha ranging from 427–893 ha; Aguiar et al. 2014). Therefore, considering these behavioral characteristics and diversification patterns, the genus *Micronycteris* represents an excellent biological model to study how the natural history of lineages influences evolutionary patterns.

An additional factor that could have influenced the lineage differentiation within the genus *Micronycteris* is the Andean Cordillera. In general, the Andes have impacted the diversification of lowland mammal fauna, inducing basal splits into trans-Andean and cis-Andean components (Patterson et al. 2012) as is seen in *M. hirsuta*, *M. minuta*, and *M. schmidtorum* complexes. Furthermore, the altitudinal ranges of the Andes have been associated with the diversification of several lineages of other genera of Phyllostomid bats such as *Platyrrhinus* (Velazco and Patterson 2008) and *Sturnira* (Velazco and Patterson 2013). Only one clade within *M. megalotis* complex in our complete dataset represented an Andean population (2000 m a.s.l.) corresponding to a confirmed candidate species (Me G; Fig. 2), suggesting that a linage diversification in the altitudinal ranges of *Micronycteris* is plausible. Notwithstanding, more information is needed to test this hypothesis.

### Unknown evolutionary lineages

The assessment of species diversity within *Micronycteris* is a complex task due to the large morphological variation and a lack of precision in the assignation of genetic sequences to specific lineages. Our analyses identified several independently evolving evolutionary lineages that correspond to different species and could be described under an integrative taxonomic approach. Currently, 13 species are accepted (Siles and Baker 2020); but considering the high number of confirmed candidate species (CCS) discussed here, we estimate that the genus comprises at least 27 species. Nevertheless, despite our comprehensive data set there are still geographic gaps that need to be filled in the future, including Central America, Central Amazonia, and the Andean Cordillera.

Several authors have suggested that the genus *Micronycteris* includes high cryptic diversity (e.g., Porter et al. 2007). However, the assessment of cryptic diversity has been limited by: (1) the incongruence between mitochondrial and nuclear genetic data, (2) the use of the genetic species concept (Bradley and Baker 2001) as the principal line of evidence, and (3) the variability of morphological data. For example, Clare (2011) in a search for cryptic species revealed incongruence between the mitochondrial gene (COI) and a region of paternally inherited Y-chromosome (Dby ^7th^ intron) in *M. megalotis*. The incongruence between markers was due to the slower divergence of the Y-chromosome region that could reflect incomplete lineage sorting in that gene (Clare 2011). However, our analyses revealed unique haplotypes in the Fgb-I7, matching most of the clades revealed by the mtDNA data analyses.

In another context, the use of genetic distance for species delimitation within *Micronycteris* (the genetic species concept) has not been homogeneously employed in the literature. For example, Clare (2011) proposes that *M. megalotis* is a species with a high intraspecific genetic variation that is the highest reported between recognized bats (COI: mean = 4.2%, range 0–7.7% using Kimura 2 parameter). Nevertheless, some authors have used smaller genetic values (near 2% in the Cytb) to identify and describe new species (e.g., *M. buriri* by Larsen et al. 2011). The genetic distance as the only criterion for defining species must be used with caution because such a measure is generally based on single mitochondrial genes with relatively high mutational rates like the Cytb (Bradley and Baker 2001). In our study, we used a conservative approach where the genetic distance between our identified genealogical lineages that correspond to undescribed species ranged above 3.0%, except for *M. buriri* and M.*matses* that differed between 1.90% and 2.90% respectively from its most closely related lineage.

Finally, *Micronycteris* has had a complex morphological taxonomy for multiple reasons. Several of the recognized species were described based on morphological characters that, after being described, were re-evaluated or redefined posterior to the revision to a higher number of specimens. That was the case of *M. homezorum* and *M. minuta* (Ochoa and Sánchez 2005), *M. minuta* and *M. sanborni* (Feijó et al. 2015), and *M. microtis* and *M. megalotis* (Martins et al. 2014). Furthermore, the description of several species was based on the morphological revision of a few specimens from a few localities. This was the case of *M. matses* (eight specimens from only one locality; Simmons et al. 2002), *M. giovanniae* (one specimen from only one locality; Fonseca et al. 2009), *M. yatesi* (three specimens from only three localities; Siles et al. 2013) and *M. buriri* (19 specimens for San Vicente Island; Larsen et al. 2011). This problematic taxonomic scenario weakens the validity of several species of the genus due to the lack of discrete morphological characters, limited knowledge on morphological variation, and the use of the phylogenetic species concept based on a single locus and the small interspecific genetic distances of differentiation (see Bradley and Baker 2001; Zachos et al. 2013). Taking those problems into account and based on our results, in the following section we clarify some aspects concerning essential issues needed for taxonomic stability in the genus *Micronycteris*, and we propose some lines of research that should be considered for future exploration.

### Taxonomic considerations

#### Subgenus Schizonycteris

A previous phylogenetic hypothesis showed *M. minuta* as paraphyletic (Siles et al. 2013). In our analyses, *M. minuta* was shown to be monophyletic, maintaining a high genetic divergence between its seven independently evolving evolutionary lineages (Suppl. material 3: Table S2). Consequently, by using integrative taxonomic methods combining molecular and robust morphological data, the identified evolutionary lineages within *M. minuta* should be described as new species. Furthermore, it is crucial to perform a broad geographic sampling throughout the distributional range of this species complex to reveal its real diversity. An additional critical issue is the assignation of the available names for each clade within the *M. minuta* complex. Nominal species has type locality in Bahia, eastern Brazil, but not sequences are included in our analyses near to that locality. Another two junior synonyms are *M. hypoleuca* from Santa Marta, Colombia (Northern Colombia; Allen 1900), and *M. homezorum* from Zulia, Venezuela (Pirlot 1967). Considering our hypothesized distributions, the lineage Mi C is distributed near these two type localities. Therefore, additional genetic information from museums or type localities is required to resolve the validity of these synonyms.

*Micronycteris yatesi*, *M. tresamici*, and *M. simmonsae* were supported in all our analyses performed with the Cytb gene and nuclear data confirming as valid species. Contrarily, it was not possible to include *M. sanborni* in our analyses because the only sequence available corresponded to a partial sequence and a chimera of individuals from Brazil (see Siles et al. 2013); and no other sequences from Brazil are available for comparisons. Recently, the morphological characters that differentiated *M. minuta* and *M. sanborni* were revalidated based on comparisons of multiple sets of specimens assignable to both taxa (Feijó et al. 2015). Because of these problems, we cannot conclude that *M. sanborni* is valid. However, the validity of *M. sanborni* should be assessed by analyzing molecular and robust morphological data, including the complete cryptic diversity of *M. minuta*, morphologically most similar species.

*Micronycteris schmidtorum* (Sanborn, 1935). In this study, *M. schmidtorum* was recovered within the subgenus Leuconycteris and as a sister species of *M. brosseti*. This result contrasts other phylogenetic hypotheses that placed the species into the *M. minuta* clades within the *Schizonycteris* subgenus (Porter et al. 2007; Siles et al. 2013) but agrees with the most recent phylogenetic assessments of *Micronycteris* (Baker and Siles 2020). The position of *M. schmidtorum* of the subgenus Leuconycteris was recently reported by Siles and Baker (2020) providing a morphological redescription of both subgenus Schizonycteris and *Leuconycteris*. The previous inclusion of *M. schmidtorum* in the subgenus Schizonycteris was due to sequences misidentification. Previous sequences used in molecular phylogenetics were: (1) a specimen from Peru housed in the American Museum of Natural History (AMNH 273172) and preserved in ethanol. After a careful morphological revision made by DMMM, this specimen was confirmed as corresponding to *M. matses*. (2) one specimen reported by Porter et al. (2007) from Bolivia, Santa Cruz, National Park Noel Kempff Mercado (without an associated voucher specimen) was recently excluded from the most recent Bolivian mammal checklist (Aguirre et al. 2019), and (3) three specimens mentioned in the phylogenetic hypothesis of Larsen et al. (2011) as M. cf. schmidtorum were genetically indistinguishable from *M. minuta* for both the Cytb and Fgb-I7 genes, a situation also observed by Siles and Baker (2020).

The individuals of *M. schmidtorum* are commonly misidentified as *M. megalotis* and *M. minuta* (Morales-Martínez et al. 2018). However, all individuals of *M. schmidtorum* included in this research and morphologically identified by DMMM were genetically distinct from *M. megalotis* and *M. minuta*. *Micronycteris schmidtorum* has the following set of morphological characters that are similar to *M. brosseti* and are completely distinguishable from species of the subgenus Schizonycteris (*M. minuta*, *M. tresamici*, *M. sanborni*, *M. simmonsae*, and *M. yatesi*): (1) the second phalange of the IV digit is shorter than the first (similar in length in *Schizonycteris* species), (2) the calcar is longer than the foot (is shorter or similar in length in *Schizonycteris* species), the mastoid width is shorter than the zygomatic width (the mastoid width is greater than zygomatic width in *Schizonycteris* species) and (3) p3 is slightly shorter than p2 and p4 (p3 much shorter than p2 and p4 in *Schizonycteris* species).

Our phylogenetic analyses and the presence of unique nuclear haplotypes matching mtDNA haplotypes showed that *M. schmidtorum* was comprised of three lineages from both sides of the eastern Andean Cordillera and French Guiana. However, considering the geographical gaps of our assessment (Central America and Amazon) the genetic diversity could be greater. On the other hand, the genetic divergence for the three lineages for the Cytb gene was low, between 1.8% and 2.0%, being smaller than the Genetic Species Concept values (ca. 4%; Baker and Bradley 2006) suggesting that no cryptic diversity is expected in *M. schmidtorum*. Nevertheless, Morales-Martínez et al. (2018) found morphometric differences between individuals of several countries and hypothesized that the Andes Cordillera and a possible cryptic diversity could explain that variation. Therefore, we suggest an assessment of those hypotheses using an integrative taxonomic approach including more individuals from different localities on both sides of the Andes.

*Micronycteris hirsuta* (Peters, 1869) was recovered as a species that exhibited a considerable high genetic variation; with 7.8% differentiation in the Cytb gene between its two forming clades (Suppl. material 3: Table S2). This value is higher than the values of interspecific variation reported for mammals in Bradley and Baker (2001). Additionally, these two clades were recognized by all species delimitation procedures, and they probably agreed with cytogenetic information. Ribas et al. (2013) reported that *M. hirsuta* has different karyotypic arrangements that do not represent a monotypic taxon, principally between the karyotypes of individuals from the Amazon in Brazil and western Ecuador. The analyzed data of Ribas et al. (2013) for the Amazon in Brazil were taken from individuals that came from localities far away from the Amazonian localities of the individuals included in this study. Although there is a direct correlation between the karyotypic differentiation and our molecular systematics results for *M. hirsuta*, we should be cautious because we cannot corroborate that the Amazonian individuals correspond to the lineage Hi A recovered by us.

*Micronycteris matses* Simmons, Voss & Fleck, 2002. *Micronycteris matses* is a species described based on eight specimens for one locality in the Peruvian Amazon (Simmons et al. 2002). The main differences that separate this species are based on the body size of the type series. This species was not shown to be a different evolutionary lineage using genetic distances because it showed less than 3% of divergence in the Cytb gene compared to *M. megalotis* (Me 1), but its distinction was recovered in all applied delimitation methods. We consider *M. matses* to be a valid species because it is validated by delimitation analyses, it presents unique nDNA haplotypes matching mtDNA haplotypes, and morphologically the cranium is more robust than in other clades of the *M. megalotis* species group (see Simmons et al. 2002).

*Micronycteris microtis* Miller, 1898. Simmons and Voss (1998) proposed a set of characters that differentiate *M. microtis* from *M. megalotis*, including the size of the ears and the length of the hairs in the anterior surface of the pinna. In contrast, Martins et al. (2014) described the variation of *M. microtis* and *M. megalotis* from Brazil and showed a superposition of the ear length with the length of the hairs of the pinna as the unique diagnostic character for distinguishing these two species. Porter et al. (2007) included specimens of the two species from Paracou (French Guiana) as determined by Simmons and Voss (1998) in their phylogenetic study, revealing those individuals as part of the same clade (clade D in Porter et al. 2007). Identifications of specimens from Colombia based on hair characters as *M. microtis* and *M. megalotis* sequenced by us, also appeared in the same clade (*M. megalotis* Me H in this study). This situation proved that such morphometric characters cannot distinguish between both species and suggested that both species may be part of the intraspecific variation within several lineages of the *M. megalotis* complex. Posteriorly to Porter et al. (2007) hypothesis, *M. microtis* was included in Larsen et al. (2011) and Siles et al. (2013) as a clade in the middle of *M. megalotis* complex apparently due to the identification of the specimen ROM 111099 gathered from GenBank from Brazil (AY380755; Me B in this work). We do not recommend considering *M. microtis* and *M. megalotis* as distinct species and suggest including them both as *M. megalotissensu lato* until a careful assessment of the diversity within the *M. megalotis* species complex is accomplished. If after a careful taxonomic revision of the *M. megalotis* complex *M. microtis* proven to be valid, that lineage should correspond to Me F or Me H clades of this study, based on the type locality in Central America (Greytown [= San Juan del Norte], Nicaragua; Miller 1898).

*Micronycteris buriri* Larsen, Siles, Pedersen & Kwiecinski, 2011. This species fails the genetic divergence limits for the Cytb gene but is recovered in two species delimitation models and has unique nDNA haplotypes. Additionally, its body size is distinguishable from other species of *Micronycteris* confirming as a valid species despite its low genetic divergence (< 2%) with other clades within the *M. megalotis* complex lineages. Other morphological characters of this species must be taken cautiously. For example, the interpretation of the lower hypsodont incisors in *M. buriri* is incorrect. This character is defined as: “the heights of crowns are roughly three times their widths” in *M. hirsuta* (Simmons 2002: fig. 3, p 9). This condition is not present in *M. buriri*, according to the images presented by Larsen et al. (2011, plate 3, p 699). Additionally, those comparisons were based on few specimens, omitting the wide morphological variation within all clades of *M. megalotis* complex.

*Micronycteris megalotis* species group. This species group (including *M. microtis*) varied extensively in the delimitation analyses. However, our research revealed at least nine genealogical lineages that are supported by most of the analyses. Several of those lineages are allopatric, parapatric, and sympatric, with two or three polyphyletic clades co-occurring in the same locality. For example, some individuals of the lineages Me C, Me E and Me H were collected at the same locality in San José del Guaviare, Guaviare Department, Colombia, and these individuals showed differences in size (see Morales-Martínez 2017). Clare (2011) proposed that the presence of polyphyletic clades with sympatric ranges is evidence that speciation is occurring in *Micronycteris*. For a posterior assessment of cryptic diversity and a description of the different evolutionary lineages representing different species, we recommend using integrative taxonomy including multiple genes mtDNA and nDNA molecular data, as well as other lines of evidence like morphology, bioacoustics, and cytogenetics.

## Supplementary materials

Supplementary material 1 Table S1. GenBank accession numbers of sequences included and specimens examined, its specimen number, and source of the sequences Data type molecular data Explanation note Specimen with an asterisk represent specimens revised by authors to confirm identifications. List of collections acronym that appear in table: American Museum of Natural History (AMNH); Carnegie Museum of Natural History (CMNH); Colección Boliviana de Fauna (CBF); Museo de Historia Natural Alcide d'Orbigny (MHNC–M); Museum of Southwestern Biology (MSB–catalog number, NK–tissue number); Museum of Texas Tech University (TTU–voucher number, TK–tissue number); Pontificia Universidad Católica del Ecuador (QCAZ); Royal Ontario Museum (ROM); United States National Museum of Natural History (NMNH); Instituto de Ciencias Naturales-Universidad Nacional de Colombia (ICN); EAFIT University, Colombia (EAFIT); Universidad Industrial de Santander (UIS). Temp: Specimens deposited in collection but uncatalogued. Boldface identifies sequences used in the delimitation analyses. Acronyms of countries: Arg: Argentina, Bel: Belize, Bol: Bolivia, Bra: Brazil, Col: Colombia, CRic: Costa Rica, Ecu: Ecuador, FGui: French Guiana, Gua: Guatemala, Guy: Guyana, Hon: Honduras, Mex: Mexico, Nic: Nicaragua, Pan: Panama, Per: Peru, Sur: Suriname, SVin: Saint Vincent and the Grenadines, Tri: Trinidad and Tobago, Ven: Venezuela.. https://binary.pensoft.net/file/528912This dataset is made available under the Open Database License (http://opendatacommons.org/licenses/odbl/1.0/). The Open Database License (ODbL) is a license agreement intended to allow users to freely share, modify, and use this Dataset while maintaining this same freedom for others, provided that the original source and author(s) are credited.Darwin M. Morales-Martínez, Hugo F. López-Arévalo, Mario Vargas-Ramírez

Supplementary material 2 Figure S1. Geographic location of sequences used in molecular analyses Data type ocurrences https://binary.pensoft.net/file/528913This dataset is made available under the Open Database License (http://opendatacommons.org/licenses/odbl/1.0/). The Open Database License (ODbL) is a license agreement intended to allow users to freely share, modify, and use this Dataset while maintaining this same freedom for others, provided that the original source and author(s) are credited.Darwin M. Morales-Martínez, Hugo F. López-Arévalo, Mario Vargas-Ramírez

Supplementary material 3 Tables S2–S2.4 Data type molecular data Explanation note **Table S2.** Average *p*-genetic distances within (diagonal) and between (below the diagonal) species and clades per subgenus of *Micronycteris* based on the Cytb gene. Acronyms represent the lineages of *M. megalotis*, *M. hirsuta* and *M. minuta* species complex. **Table S2.1.** Subgenus Micronycteris. **Table S2.2.** Subgenus Xenonectes. **Table S2.3.** Subgenus Leuconycteris. **Table S2.4.** Subgenus Schizonycteris. https://binary.pensoft.net/file/528914This dataset is made available under the Open Database License (http://opendatacommons.org/licenses/odbl/1.0/). The Open Database License (ODbL) is a license agreement intended to allow users to freely share, modify, and use this Dataset while maintaining this same freedom for others, provided that the original source and author(s) are credited.Darwin M. Morales-Martínez, Hugo F. López-Arévalo, Mario Vargas-Ramírez

Supplementary material 4 Results of the delimitation analyses Data type Species delimitation analyses https://binary.pensoft.net/file/528915This dataset is made available under the Open Database License (http://opendatacommons.org/licenses/odbl/1.0/). The Open Database License (ODbL) is a license agreement intended to allow users to freely share, modify, and use this Dataset while maintaining this same freedom for others, provided that the original source and author(s) are credited.Darwin M. Morales-Martínez, Hugo F. López-Arévalo, Mario Vargas-Ramírez
