# Supplementary material for: Beginning the quest: phylogenetic hypothesis and identification of evolutionary lineages in bats of the genus Micronycteris (Chiroptera, Phyllostomidae)
Source: Zookeys. 2021 Apr 6;1028:135–59. doi: 10.3897/zookeys.1028.60955 (PMC8044067; doi:10.3897/zookeys.1028.60955)
Supplement: Supplementary material 1 — Table S1. GenBank accession numbers of sequences included and specimens examined, its specimen number, and source of the sequences [file zookeys-1028-135-s001.docx]

Supplementary information

Table S1.

GenBank accession numbers of sequences analyzed and specimens examined in this study. Specimens revised by authors to confirm identifications marked with an asterisk. Collections acronyms: American Museum of Natural History (**AMNH**); Carnegie Museum of Natural History (**CMNH**); Colección Boliviana de Fauna (**CBF**); Museo de Historia Natural Alcide d'Orbigny (**MHNC–M**); Museum of Southwestern Biology (**MSB**–catalog number, **NK**–tissue number); Museum of Texas Tech University (**TTU**–voucher number, **TK**–tissue number); Pontificia Universidad Católica del Ecuador (**QCAZ**); Royal Ontario Museum (**ROM**); United States National Museum of Natural History (**NMNH**); Instituto de Ciencias Naturales-Universidad Nacional de Colombia (**ICN**); EAFIT University, Colombia (**EAFIT**); Universidad Industrial de Santander (**UIS**). Temp: Specimens deposited in collection but uncatalogued. Sequences used in the delimitation analyses in bold. Acronyms of countries: Arg: Argentina, Bel: Belize, Bol: Bolivia, Bra: Brazil, Col: Colombia, CRic: Costa Rica, Ecu: Ecuador, FGui: French Guiana, Gua: Guatemala, Guy: Guyana, Hon: Honduras, Mex: Mexico, Nic: Nicaragua, Pan: Panama, Per: Peru, Sur: Suriname, SVin: Saint Vincent and the Grenadines, Tri: Trinidad and Tobago, Ven: Venezuela.

| Subgenus | Species | clade | Voucher | Cytb | Fgb-I7 | Country | Latitud | Longitud | Source |
| --- | --- | --- | --- | --- | --- | --- | --- | --- | --- |
| *Leuconycteris* | *brosseti* | brosseti | **KU155162** | AY380770 | DQ077454 | Guy | 4.63 | -58.72 | Porter et al. (2007) |
| *Leuconycteris* | *brosseti* | brosseti | **KU155163** | AY380771 | DQ077455 | Guy | 4.63 | -58.72 | Porter et al. (2007) |
| *Leuconycteris* | *schmidtorum* | schmidtorum | **AMNH267854** * | MN707463 | MN707482 | FGui | 5.28 | -52.92 | Siles & Baker (2020) |
| *Leuconycteris* | *schmidtorum* | schmidtorum | **TTU103196** | MN707462 | MN707478 | Ecu | 1.07 | -78.71 | Siles & Baker (2020) |
| *Leuconycteris* | *schmidtorum* | schmidtorum | **ICN 24479 *** | MK936333 | MK936367 | Col | 11.28 | -74.15 | This study |
| *Leuconycteris* | *schmidtorum* | schmidtorum | **ICN 21576 *** | MK936330 | MK936368 | Col | 3.72 | -73.26 | This study |
| *Leuconycteris* | *schmidtorum* | schmidtorum | **ICN 22940 *** | MK936331 |  | Col | 5.44 | -71.76 | This study |
| *Schizonycteris* | *tresamici* | tresamici | **TTU61057** | MN707448 | MN707490 | Hon | 15.70 | -87.47 | Siles & Baker (2020) |
| *Schizonycteris* | *tresamici* | tresamici | **TTU103880** | MN707449 | MN707480 | Hon | 15.73 | -87.46 | Siles & Baker (2020) |
| *Schizonycteris* | *minuta* | Mi A | **ICN 24465 *** | MK936353 | MT380813 | Col | 1.51 | -75.67 | This study |
| *Schizonycteris* | *minuta* | Mi A | **ROM104067** | AY380752 | DQ077438 | Ecu | -0.62 | -76.47 | Porter et al. (2007) |
| *Schizonycteris* | *minuta* | Mi A | **QCAZ6299 * (TTU84825)** | DQ077404 | DQ077439 | Ecu | -1.47 | -77.98 | Porter et al. (2007) |
| *Schizonycteris* | *minuta* | Mi B | **UIS Temp JEC 193** | MT360270 |  | Col | 7.38 | -73.93 | This study |
| *Schizonycteris* | *minuta* | Mi B | **ICN 25302** | MK936336 |  | Col | 5.84 | -72.17 | This study |
| *Schizonycteris* | *minuta* | Mi B | **ICN Temp DRG 086 *** | MK936339 | MK936366 | Col | 5.84 | -72.17 | This study |
| *Schizonycteris* | *minuta* | Mi B | **ICN 22939 *** | MK936339 |  | Col | 5.44 | -71.76 | This study |
| *Schizonycteris* | *minuta* | Mi B | **ICN Temp FSC 171 *** | MK936341 |  | Col | 5.44 | -71.76 | This study |
| *Schizonycteris* | *minuta* | Mi B | **ICN 24472 *** | MK936337 | MK936365 | Col | 3.59 | -73.52 | This study |
| *Schizonycteris* | *minuta* | Mi B | **ICN 23912 *** | MK936347 |  | Col | 4.42 | -68.21 | This study |
| *Schizonycteris* | *minuta* | Mi B | **TTU43946** | MN707425 | MN707501 | Tri | 10.43 | -61.06 | Siles & Baker (2020) |
| *Schizonycteris* | *minuta* | Mi B | **CM97184** | MN707424 | MN707487 | Tri | 10.72 | -61.30 | Siles & Baker (2020) |
| *Schizonycteris* | *minuta* | Mi B | TTU33280 | MN707430 | MN707499 | Ven | 8.52 | -67.43 | Siles & Baker (2020) |
| *Schizonycteris* | *minuta* | Mi B | **TTU33282** | MN707426 | MN707500 | Ven | 8.52 | -67.43 | Siles & Baker (2020) |
| *Schizonycteris* | *minuta* | Mi B | TTU33283 | MN707427 | MN707484 | Ven | 8.52 | -67.43 | Siles & Baker (2020) |
| *Schizonycteris* | *minuta* | Mi B | TTU33281 | MN707431 | MN707502 | Ven | 8.52 | -67.43 | Siles & Baker (2020) |
| *Schizonycteris* | *minuta* | Mi B | **TTU33278** | MN707428 | - | Ven | 8.52 | -67.43 | Siles & Baker (2020) |
| *Schizonycteris* | *minuta* | Mi B | TTU33284 | MN707429 | - | Ven | 8.52 | -67.43 | Siles & Baker (2020) |
| *Schizonycteris* | *minuta* | Mi C | **MSB235221** | DQ077406 | DQ077442 | Bol | -14.64 | -60.73 | Porter et al. (2007) |
| *Schizonycteris* | *minuta* | Mi C | **MHNC-M175** | MN707443 | MN707489 | Bol | -13.27 | -63.70 | Siles & Baker (2020) |
| *Schizonycteris* | *minuta* | Mi C | **MHNC-M186** | MN707444 | - | Bol | -17.40 | -64.23 | Siles & Baker (2020) |
| *Schizonycteris* | *minuta* | Mi D | **AMNH267874 *** | MN707445 | MN707488 | FGui | 5.28 | -52.92 | Siles & Baker (2020) |
| *Schizonycteris* | *minuta* | Mi D | **AMNH267875 *** | MN707446 | - | FGui | 5.28 | -52.92 | Siles & Baker (2020) |
| *Schizonycteris* | *minuta* | Mi D | **CM76769** | MN707447 | MN707483 | Sur | 5.48 | -54.07 | Siles & Baker (2020) |
| *Schizonycteris* | *minuta* | Mi E | **TTU109879** | MN707438 | MN707498 | Sur | 3.79 | -56.15 | Siles & Baker (2020) |
| *Schizonycteris* | *minuta* | Mi E | **CM77111** | MN707439 | - | Sur | 2.03 | -56.13 | Siles & Baker (2020) |
| *Schizonycteris* | *minuta* | Mi E | CM77114 | MN707440 | MN707497 | Sur | 2.03 | -56.13 | Siles & Baker (2020) |
| *Schizonycteris* | *minuta* | Mi E | **CM77112** | MN707441 | MN707495 | Sur | 2.03 | -56.13 | Siles & Baker (2020) |
| *Schizonycteris* | *minuta* | Mi E | **TTU106007** | MN707436 | - | Sur | 4.28 | -54.74 | Siles & Baker (2020) |
| *Schizonycteris* | *minuta* | Mi E | TTU106008 | MN707437 | MN707496 | Sur | 4.28 | -54.74 | Siles & Baker (2020) |
| *Schizonycteris* | *minuta* | Mi E | CM77113 | MN707442 | - | Sur | 2.03 | -56.13 | Siles & Baker (2020) |
| *Schizonycteris* | *minuta* | Mi F | **USNM582262** | AY380754 | DQ077441 | Guy | 5.68 | -57.86 | Porter et al. (2007) |
| *Schizonycteris* | *minuta* | Mi F | **USNM582263** | MN707432 | MN707485 | Guy | 5.68 | -57.86 | Siles & Baker (2020) |
| *Schizonycteris* | *minuta* | Mi F | **CM63584** | MN707433 | MN707479 | Sur | 4.68 | -56.18 | Siles & Baker (2020) |
| *Schizonycteris* | *minuta* | Mi F | **CM68639** | MN707435 | MN707494 | Sur | 4.83 | -57.23 | Siles & Baker (2020) |
| *Schizonycteris* | *minuta* | Mi F | CM68391 | MN707434 | - | Sur | 4.68 | -56.18 | Siles & Baker (2020) |
| *Schizonycteris* | *simmonsae* | simmonsae | **QCAZ9136* (TTU103253)** | DQ077400 | DQ077434 | Ecu | -2.18 | -80.02 | Porter et al. (2007) |
| *Schizonycteris* | *simmonsae* | simmonsae | QCAZ 9137* | DQ077401 | DQ077435 | Ecu | -2.18 | -80.03 | Porter et al. (2007) |
| *Schizonycteris* | *simmonsae* | simmonsae | TTU103201 | DQ077402 | DQ077436 | Ecu | 1.07 | -78.71 | Porter et al. (2007) |
| *Schizonycteris* | *simmonsae* | simmonsae | **TTU103198** | DQ077403 | DQ077437 | Ecu | 1.07 | -78.71 | Porter et al. (2007) |
| *Schizonycteris* | *yatesi* | yatesi | **MHNC-M157** | KC756212 | MN707481 | Bol | -18.78 | -65.13 | Siles & Baker (2020) |
| *Schizonycteris* | *yatesi* | yatesi | **MHNC-M141** | KC756211 |  | Bol | -18.11 | -63.61 | Siles & Baker (2020) |
| *Schizonycteris* | *yatesi* | yatesi | **CBF6154** | KC756213 |  | Bol | -17.62 | -59.50 | Siles & Baker (2020) |
| *Micronycteris* | *buriri* | buriri | TTU105353 | HQ593848 |  | SVin | 13.31 | -61.21 | Siles & Baker (2020) |
| *Micronycteris* | *buriri* | buriri | **TTU105641** | HQ593841 |  | SVin | 13.31 | -61.21 | Siles & Baker (2020) |
| *Micronycteris* | *buriri* | buriri | **TTU105642** | HQ593852 |  | SVin | 13.31 | -61.21 | Siles & Baker (2020) |
| *Micronycteris* | *buriri* | buriri | **TTU105535** | HQ593845 | MN707466 | SVin | 13.31 | -61.21 | Siles & Baker (2020) |
| *Micronycteris* | *buriri* | buriri | TTU105473 | HQ593847 | MN707493 | SVin | 13.22 | -61.21 | Siles & Baker (2020) |
| *Micronycteris* | *buriri* | buriri | **TTU105352** | HQ593840 | MN707492 | SVin | 13.32 | -61.17 | Siles & Baker (2020) |
| *Micronycteris* | *buriri* | buriri | TTU105354 | HQ593849 | MN707477 | SVin | 13.32 | -61.17 | Siles & Baker (2020) |
| *Micronycteris* | *buriri* | buriri | TTU105773 | HQ593843 | MN707467 | SVin | 13.22 | -61.21 | Siles & Baker (2020) |
| *Micronycteris* | *buriri* | buriri | TTU105971 | HQ593846 | MN707465 | SVin | 13.25 | -61.21 | Siles & Baker (2020) |
| *Micronycteris* | *buriri* | buriri | **TTU105972** | HQ593842 | MN707469 | SVin | 13.25 | -61.21 | Siles & Baker (2020) |
| *Micronycteris* | *buriri* | buriri | TTU105981 | HQ593839 | MN707468 | SVin | 13.22 | -61.28 | Siles & Baker (2020) |
| *Micronycteris* | *buriri* | buriri | TTU105982 | HQ593844 | MN707491 | SVin | 13.22 | -61.28 | Siles & Baker (2020) |
| *Micronycteris* | *giovanniae* | giovanniae | **QCAZ7200 *** | AY380750 | DQ077456 | Ecu | 1.06 | -78.62 | Porter et al. (2007) |
| *Micronycteris* | *matses* | matses | **AMNH272814 *** | DQ077417 | DQ077457 | Per | -5.20 | -72.88 | Porter et al. (2007) |
| *Micronycteris* | *matses* | matses | AMNH273043 * | DQ077418 | DQ077458 | Per | -5.20 | -72.88 | Porter et al. (2007) |
| *Micronycteris* | *matses* | matses | **AMNH273095 *** | DQ077419 | DQ077459 | Per | -5.20 | -72.88 | Porter et al. (2007) |
| *Micronycteris* | *matses* | matses | **AMNH273044 *** | MN707464 | MN707486 | Per | -5.20 | -72.88 | Porter et al. (2007) |
| *Micronycteris* | *megalotis* | Me A | **AMNH267090 *** | AY380761 | DQ077465 | FGui | 5.28 | -52.92 | Porter et al. (2007) |
| *Micronycteris* | *megalotis* | Me A | **AMNH267097 *** | AY380756 | DQ077461 | FGui | 5.28 | -52.92 | Porter et al. (2007) |
| *Micronycteris* | *megalotis* | Me A | **AMNH267868 *** | Siles & Baker (2020) | Siles & Baker (2020) | FGui | 5.28 | -52.92 | Siles & Baker (2020) |
| *Micronycteris* | *megalotis* | Me A | **AMNH267867 *** | Siles & Baker (2020) | Siles & Baker (2020) | FGui | 5.28 | -52.92 | Siles & Baker (2020) |
| *Micronycteris* | *megalotis* | Me A | **AMNH267864 *** | Siles & Baker (2020) | Siles & Baker (2020) | FGui | 5.28 | -52.92 | Siles & Baker (2020) |
| *Micronycteris* | *megalotis* | Me A | AMNH267863 * | Siles & Baker (2020) | Siles & Baker (2020) | FGui | 5.28 | -52.92 | Siles & Baker (2020) |
| *Micronycteris* | *megalotis* | Me A | **AMNH267865 *** | Siles & Baker (2020) | Siles & Baker (2020) | FGui | 5.28 | -52.92 | Siles & Baker (2020) |
| *Micronycteris* | *megalotis* | Me A | **AMNH267866 *** | Siles & Baker (2020) | Siles & Baker (2020) | FGui | 5.28 | -52.92 | Siles & Baker (2020) |
| *Micronycteris* | *megalotis* | Me A | **AMNH267862** * | Siles & Baker (2020) | Siles & Baker (2020) | FGui | 5.28 | -52.92 | Siles & Baker (2020) |
| *Micronycteris* | *megalotis* | Me A | AMNH267869 * | Siles & Baker (2020) | Siles & Baker (2020) | FGui | 5.28 | -52.92 | Siles & Baker (2020) |
| *Micronycteris* | *megalotis* | Me A | **ROM108745** | AY380757 | DQ077462 | Guy | 4.33 | -58.80 | Porter et al. (2007) |
| *Micronycteris* | *megalotis* | Me A | **CM63575** | Siles & Baker (2020) | Siles & Baker (2020) | Sur | 3.78 | -58.16 | Siles & Baker (2020) |
| *Micronycteris* | *megalotis* | Me A | **CM63577** | Siles & Baker (2020) | Siles & Baker (2020) | Sur | 3.81 | -58.15 | Siles & Baker (2020) |
| *Micronycteris* | *megalotis* | Me A | CM68390 | AY380758 | DQ077464 | Sur | 3.09 | -56.48 | Porter et al. (2007) |
| *Micronycteris* | *megalotis* | Me A | **CM76768** | AY380759 | DQ077466 | Sur | 5.67 | -54.45 | Porter et al. (2007) |
| *Micronycteris* | *megalotis* | Me A | TTU109878 | Siles & Baker (2020) | Siles & Baker (2020) | Sur | 4.72 | -56.20 | Siles & Baker (2020) |
| *Micronycteris* | *megalotis* | Me A | **CM68389** | Siles & Baker (2020) | Siles & Baker (2020) | Sur | 3.09 | -56.48 | Siles & Baker (2020) |
| *Micronycteris* | *megalotis* | Me A | CM77109 | Siles & Baker (2020) | - | Sur | 5.67 | -54.45 | Siles & Baker (2020) |
| *Micronycteris* | *megalotis* | Me A | **CM97180** | Siles & Baker (2020) | Siles & Baker (2020) | Tri | 10.43 | -61.06 | Siles & Baker (2020) |
| *Micronycteris* | *megalotis* | Me A | **CM78295** | AY380773 | DQ077467 | Ven | 7.54 | -62.35 | Porter et al. (2007) |
| *Micronycteris* | *megalotis* | Me A | CM78296 | Siles & Baker (2020) | - | Ven | 7.54 | -62.35 | Siles & Baker (2020) |
| *Micronycteris* | *megalotis* | Me A | **CM78294** | Siles & Baker (2020) | Siles & Baker (2020) | Ven | 7.54 | -62.35 | Siles & Baker (2020) |
| *Micronycteris* | *megalotis* | Me A | **CM78299** | Siles & Baker (2020) | Siles & Baker (2020) | Ven | 7.97 | -61.63 | Siles & Baker (2020) |
| *Micronycteris* | *megalotis* | Me A | **CM78297** | Siles & Baker (2020) | Siles & Baker (2020) | Ven | 7.28 | -62.47 | Siles & Baker (2020) |
| *Micronycteris* | *megalotis* | Me B | **ROM111099** | AY380755 | DQ077463 | Bra | -22.38 | -49.67 | Porter et al. (2007) |
| *Micronycteris* | *megalotis* | Me C | **ICN 21568 *** | MK936328 |  | Col | 4.09 | -72.96 | This study |
| *Micronycteris* | *megalotis* | Me C | **ICN 22674 *** | MK936329 | MK936361 | Col | 2.59 | -72.61 | This study |
| *Micronycteris* | *megalotis* | Me D | **CML7552** | Siles & Baker (2020) | - | Arg | -22.73 | -64.36 | Siles & Baker (2020) |
| *Micronycteris* | *megalotis* | Me D | **CML7553** | Siles & Baker (2020) | Siles & Baker (2020) | Arg | -22.73 | -64.36 | Siles & Baker (2020) |
| *Micronycteris* | *megalotis* | Me D | **MHNC-M189** | Siles & Baker (2020) | - | Bol | -17.40 | -64.23 | Siles & Baker (2020) |
| *Micronycteris* | *megalotis* | Me D | **MHNC-M144** | Siles & Baker (2020) | - | Bol | -16.67 | -66.48 | Siles & Baker (2020) |
| *Micronycteris* | *megalotis* | Me D | **MUSM13210** | Siles & Baker (2020) | Siles & Baker (2020) | Per | -5.20 | -72.88 | Siles & Baker (2020) |
| *Micronycteris* | *megalotis* | Me D | **AMNH273081 *** | Siles & Baker (2020) | Siles & Baker (2020) | Per | -5.20 | -72.88 | Siles & Baker (2020) |
| *Micronycteris* | *megalotis* | Me D | **AMNH273098 *** | Siles & Baker (2020) | Siles & Baker (2020) | Per | -5.20 | -72.88 | Siles & Baker (2020) |
| *Micronycteris* | *megalotis* | Me D | **AMNH273169 *** | DQ077407 | DQ077443 | Per | -5.20 | -72.88 | Porter et al. (2007) |
| *Micronycteris* | *megalotis* | Me D | **AMNH273072 *** | Siles & Baker (2020) | Siles & Baker (2020) | Per | -5.20 | -72.88 | Siles & Baker (2020) |
| *Micronycteris* | *megalotis* | Me E | **ICN 23839 *** | MK936353 | MT380812 | Col | 2.59 | -72.61 | This study |
| *Micronycteris* | *megalotis* | Me F | **AMNH274585 *** | Siles & Baker (2020) | Siles & Baker (2020) | Bel | 17.55 | -89.03 | Siles & Baker (2020) |
| *Micronycteris* | *megalotis* | Me F | **QCAZ9580 * (TTU102602)** | DQ077427 | DQ077478 | Ecu | -3.88 | -80.09 | Porter et al. (2007) |
| *Micronycteris* | *megalotis* | Me F | TTU103291 | DQ077428 | DQ077479 | Ecu | -2.43 | -80.02 | Porter et al. (2007) |
| *Micronycteris* | *megalotis* | Me F | TTU103437 | DQ077429 | DQ077480 | Ecu | -2.74 | -79.91 | Porter et al. (2007) |
| *Micronycteris* | *megalotis* | Me F | **TTU85289** | DQ077426 | DQ077477 | Ecu | 1.24 | -78.76 | Porter et al. (2007) |
| *Micronycteris* | *megalotis* | Me F | **QCAZ9135 * (TTU85389)** | DQ077422 | DQ077473 | Ecu | 1.27 | -78.80 | Porter et al. (2007) |
| *Micronycteris* | *megalotis* | Me F | QCAZ9134 *  (TTU85435) | DQ077424 | DQ077476 | Ecu | 1.24 | -78.76 | Porter et al. (2007) |
| *Micronycteris* | *megalotis* | Me F | TTU85436 | DQ077425 | DQ077475 | Ecu | 1.24 | -78.76 | Porter et al. (2007) |
| *Micronycteris* | *megalotis* | Me F | **TTU102918** | DQ077423 | DQ077474 | Ecu | 1.09 | -78.71 | Porter et al. (2007) |
| *Micronycteris* | *megalotis* | Me F | **TTU102398** | Siles & Baker (2020) | Siles & Baker (2020) | Ecu | -3.77 | -79.65 | Siles & Baker (2020) |
| *Micronycteris* | *megalotis* | Me F | TTU103439 | Siles & Baker (2020) | - | Ecu | -2.74 | -79.91 | Siles & Baker (2020) |
| *Micronycteris* | *megalotis* | Me F | **TTU103387** | Siles & Baker (2020) | Siles & Baker (2020) | Ecu | -2.76 | -79.92 | Siles & Baker (2020) |
| *Micronycteris* | *megalotis* | Me F | **TTU103800** | Siles & Baker (2020) | - | Ecu | -3.90 | -80.08 | Siles & Baker (2020) |
| *Micronycteris* | *megalotis* | Me F | **TTU103284** | Siles & Baker (2020) | - | Ecu | -2.43 | -80.02 | Siles & Baker (2020) |
| *Micronycteris* | *megalotis* | Me F | **TTU102448** | Siles & Baker (2020) | Siles & Baker (2020) | Ecu | -3.69 | -79.60 | Siles & Baker (2020) |
| *Micronycteris* | *megalotis* | Me F | TTU103289 | Siles & Baker (2020) | - | Ecu | -2.43 | -80.02 | Siles & Baker (2020) |
| *Micronycteris* | *megalotis* | Me F | TTU103290 | Siles & Baker (2020) | - | Ecu | -2.43 | -80.02 | Siles & Baker (2020) |
| *Micronycteris* | *megalotis* | Me F | TTU103491 | Siles & Baker (2020) | - | Ecu | -2.74 | -79.91 | Siles & Baker (2020) |
| *Micronycteris* | *megalotis* | Me F | TTU103778 | Siles & Baker (2020) | Siles & Baker (2020) | Ecu | -2.16 | -80.03 | Siles & Baker (2020) |
| *Micronycteris* | *megalotis* | Me F | **TTU103436** | Siles & Baker (2020) | - | Ecu | -2.74 | -79.91 | Siles & Baker (2020) |
| *Micronycteris* | *megalotis* | Me F | TTU103438 | Siles & Baker (2020) | Siles & Baker (2020) | Ecu | -2.74 | -79.91 | Siles & Baker (2020) |
| *Micronycteris* | *megalotis* | Me F | TTU103285 | Siles & Baker (2020) | Siles & Baker (2020) | Ecu | -2.43 | -80.02 | Siles & Baker (2020) |
| *Micronycteris* | *megalotis* | Me F | TTU85424 | Siles & Baker (2020) | - | Ecu | 1.24 | -78.76 | Siles & Baker (2020) |
| *Micronycteris* | *megalotis* | Me F | **TTU103311** | Siles & Baker (2020) | Siles & Baker (2020) | Ecu | -2.18 | -80.03 | Siles & Baker (2020) |
| *Micronycteris* | *megalotis* | Me F | **TK167801** | Siles & Baker (2020) | Siles & Baker (2020) | CRic | 10.40 | -84.11 | Siles & Baker (2020) |
| *Micronycteris* | *megalotis* | Me F | **TK167804** | Siles & Baker (2020) | - | CRic | 10.40 | -84.11 | Siles & Baker (2020) |
| *Micronycteris* | *megalotis* | Me F | TK167811 | Siles & Baker (2020) | Siles & Baker (2020) | CRic | 10.40 | -84.11 | Siles & Baker (2020) |
| *Micronycteris* | *megalotis* | Me F | **TTU36534** | AY380764 | DQ077472 | Mex | 16.03 | -93.64 | Porter et al. (2007) |
| *Micronycteris* | *megalotis* | Me F | **TK45489** | Siles & Baker (2020) | Siles & Baker (2020) | Mex | 19.04 | -101.81 | Siles & Baker (2020) |
| *Micronycteris* | *megalotis* | Me F | TK45355 | Siles & Baker (2020) | Siles & Baker (2020) | Mex | 18.93 | -101.87 | Siles & Baker (2020) |
| *Micronycteris* | *megalotis* | Me F | TK45488 | Siles & Baker (2020) | Siles & Baker (2020) | Mex | 19.04 | -101.81 | Siles & Baker (2020) |
| *Micronycteris* | *megalotis* | Me F | **TTU82624** | Siles & Baker (2020) | Siles & Baker (2020) | Mex | 16.49 | -95.89 | Siles & Baker (2020) |
| *Micronycteris* | *megalotis* | Me F | **TTU35355** | Siles & Baker (2020) | Siles & Baker (2020) | Mex | 21.99 | -99.40 | Siles & Baker (2020) |
| *Micronycteris* | *megalotis* | Me F | **TK45487** | Siles & Baker (2020) | - | Mex | 19.04 | -101.81 | Siles & Baker (2020) |
| *Micronycteris* | *megalotis* | Me F | **TK45332** | Siles & Baker (2020) | Siles & Baker (2020) | Mex | 19.98 | -101.81 | Siles & Baker (2020) |
| *Micronycteris* | *megalotis* | Me F | **TK45330** | Siles & Baker (2020) | Siles & Baker (2020) | Mex | 19.98 | -101.81 | Siles & Baker (2020) |
| *Micronycteris* | *megalotis* | Me F | **TK45354** | Siles & Baker (2020) | Siles & Baker (2020) | Mex | 18.93 | -101.87 | Siles & Baker (2020) |
| *Micronycteris* | *megalotis* | Me G | **ICN 24495 *** | MK936325 | MK936356 | Col | 2.21 | -75.65 | This study |
| *Micronycteris* | *megalotis* | Me G | **ICN 24494 *** | MK936334 |  | Col | 2.21 | -75.65 | This study |
| *Micronycteris* | *megalotis* | Me G | **ICN 23730 *** | MK936348 |  | Col | 1.74 | -75.92 | This study |
| *Micronycteris* | *megalotis* | Me G | **ICN 23731 *** | MK936349 |  | Col | 1.74 | -75.92 | This study |
| *Micronycteris* | *megalotis* | Me H | **ICN 21000 *** | MK936326 |  | Col | 4.85 | -73.00 | This study |
| *Micronycteris* | *megalotis* | Me H | **ICN 24484 *** | MK936327 | MK936357 | Col | 6.68 | -75.81 | This study |
| *Micronycteris* | *megalotis* | Me H | EAFIT JFD1311 | MACAU037-19 |  | Col | 8.03 | -75.22 | Bold Systems accesion |
| *Micronycteris* | *megalotis* | Me H | **ICN 17736 *** | MK936344 |  | Col | 9.21 | -73.50 | This study |
| *Micronycteris* | *megalotis* | Me H | **ICN 21124 *** | MK936346 | MK936358 | Col | 7.39 | -73.45 | This study |
| *Micronycteris* | *megalotis* | Me H | **ICN Temp DRG 076 *** | MK936335 | MK936360 | Col | 5.84 | -72.17 | This study |
| *Micronycteris* | *megalotis* | Me H | ICN 25097 * | MK936342 |  | Col | 5.25 | -72.89 | This study |
| *Micronycteris* | *megalotis* | Me H | **ICN 22485 *** | MK936338 |  | Col | 2.59 | -72.61 | This study |
| *Micronycteris* | *megalotis* | Me H | **ICN 23203 *** | MK936324 | MK936355 | Col | 2.25 | -73.82 | This study |
| *Micronycteris* | *megalotis* | Me H | **ROM104195** | AY380765 | DQ077468 | Pan | 9.10 | -79.70 | Porter et al. (2007) |
| *Micronycteris* | *megalotis* | Me H | **TTU33276** | AY380763 | DQ077471 | Ven | 8.52 | -67.43 | Porter et al. (2007) |
| *Micronycteris* | *megalotis* | Me H | **CM78291** | DQ077421 | DQ077469 | Ven | 7.76 | -71.23 | Porter et al. (2007) |
| *Micronycteris* | *megalotis* | Me H | **CM78298** | Siles & Baker (2020) | Siles & Baker (2020) | Ven | 7.28 | -62.47 | Siles & Baker (2020) |
| *Micronycteris* | *megalotis* | Me H | **CM78292** | Siles & Baker (2020) | Siles & Baker (2020) | Ven | 8.76 | -70.31 | Siles & Baker (2020) |
| *Micronycteris* | *megalotis* | Me I | **CM97182** | Siles & Baker (2020) | Siles & Baker (2020) | Tri | 10.72 | -61.30 | Siles & Baker (2020) |
| *Micronycteris* | *megalotis* | Me I | CM97183 | Siles & Baker (2020) | Siles & Baker (2020) | Tri | 10.72 | -61.30 | Siles & Baker (2020) |
| *Micronycteris* | *megalotis* | Me I | **TTU43944** | Siles & Baker (2020) | DQ077470.1 | Tri | 11.19 | -60.79 | Siles & Baker (2020) |
| *Micronycteris* | *megalotis* | Me I | **UNSMZM29489** | Siles & Baker (2020) | Siles & Baker (2020) | Tri | 11.29 | -60.67 | Siles & Baker (2020) |
| *Micronycteris* | *megalotis* | Me I | **UNSMZM29476** | Siles & Baker (2020) | Siles & Baker (2020) | Tri | 11.28 | -60.61 | Siles & Baker (2020) |
| *Micronycteris* | *megalotis* | Me I | UNSMZM29494 | Siles & Baker (2020) | Siles & Baker (2020) | Tri | 11.28 | -60.61 | Siles & Baker (2020) |
| *Micronycteris* | *megalotis* | Me I | **UNSMZM29493** | Siles & Baker (2020) | Siles & Baker (2020) | Tri | 11.28 | -60.61 | Siles & Baker (2020) |
| *Micronycteris* | *megalotis* | Me I | UNSMZM29491 | Siles & Baker (2020) | Siles & Baker (2020) | Tri | 11.28 | -60.61 | Siles & Baker (2020) |
| *Micronycteris* | *megalotis* | ? | ICN 12486 * |  | MT380809 | Col |  |  | This study |
| *Micronycteris* | *megalotis* | ? | ICN 21607 * |  | MK936359 | Col |  |  | This study |
| *Micronycteris* | *megalotis* | ? | ICN 9136 * |  | MT380810 | Col |  |  | This study |
| *Micronycteris* | *megalotis* | ? | ICN 17928 * |  | MT380811 | Col |  |  | This study |
| *Micronycteris* | *sp.* | M. sp. | **TTU104168** | DQ077420 | DQ077460 | Hon | 15.92 | -86.00 | Porter et al. (2007) |
| *Xenoctenes* | *hirsuta* | Hi A | **TTU85449** | DQ077410 | DQ077448 | Ecu | 1.06 | -78.62 | Porter et al. (2007) |
| *Xenoctenes* | *hirsuta* | Hi A | TTU85452 | DQ077412 | DQ077449 | Ecu | 1.06 | -78.62 | Porter et al. (2007) |
| *Xenoctenes* | *hirsuta* | Hi A | **TTU103117** | DQ077415 | DQ077453 | Ecu | 1.35 | -78.72 | Porter et al. (2007) |
| *Xenoctenes* | *hirsuta* | Hi A | **TTU85428** | DQ077414 | DQ077451 | Ecu | 1.24 | -78.76 | Porter et al. (2007) |
| *Xenoctenes* | *hirsuta* | Hi A | QCAZ9125 *  (TTU85432) | DQ077413 | DQ077450 | Ecu | 1.24 | -78.76 | Porter et al. (2007) |
| *Xenoctenes* | *hirsuta* | Hi A | **MSB94371** | AY380768 | DQ077444 | Pan | 7.24 | -80.64 | Porter et al. (2007) |
| *Xenoctenes* | *hirsuta* | Hi A | MSB94372 | AY380769 | DQ077445 | Pan | 7.24 | -80.64 | Porter et al. (2007) |
| *Xenoctenes* | *hirsuta* | Hi A | CM97177 | AY380751 | DQ077447 | Tri | 10.72 | -61.30 | Porter et al. (2007) |
| *Xenoctenes* | *hirsuta* | Hi A | TTU43943 | DQ077408 | DQ077446 | Tri | 10.13 | -61.06 | Porter et al. (2007) |
| *Xenoctenes* | *hirsuta* | Hi A | **CM68638** | MN707456 | - | Sur | 4.42 | -57.22 | Siles & Baker (2020) |
| *Xenoctenes* | *hirsuta* | Hi A | **AMNH267858** * | MN707455 | MN707475 | FGui | 5.28 | -52.92 | Siles & Baker (2020) |
| *Xenoctenes* | *hirsuta* | Hi A | TTU43942 | MN707458 | - | Tri | 10.13 | -61.06 | Siles & Baker (2020) |
| *Xenoctenes* | *hirsuta* | Hi A | **CM97178** | MN707457 | MN707473 | Tri | 10.72 | -61.30 | Siles & Baker (2020) |
| *Xenoctenes* | *hirsuta* | Hi A | **CM97176** | MN707453 | MN707472 | Tri | 10.72 | -61.30 | Siles & Baker (2020) |
| *Xenoctenes* | *hirsuta* | Hi A | **CM68387** | MN707451 | MN707474 | Sur | 5.45 | -55.20 | Siles & Baker (2020) |
| *Xenoctenes* | *hirsuta* | Hi A | CM68388 | MN707452 | MN707470 | Sur | 5.45 | -55.20 | Siles & Baker (2020) |
| *Xenoctenes* | *hirsuta* | Hi A | **AMNH267860** * | MN707454 | - | FGui | 5.28 | -52.92 | Siles & Baker (2020) |
| *Xenoctenes* | *hirsuta* | Hi A | **AMNH267857 *** | MN707450 | MN707471 | FGui | 5.28 | -52.92 | Siles & Baker (2020) |
| *Xenoctenes* | *hirsuta* | Hi A | TTU85429 | MN707461 | - | Ecu | 1.24 | -78.76 | Siles & Baker (2020) |
| *Xenoctenes* | *hirsuta* | Hi A | **TK167812** | MN707459 | - | CRic | 10.40 | -84.11 | Siles & Baker (2020) |
| *Xenoctenes* | *hirsuta* | Hi A | **TK167825** | MN707460 | MN707476 | CRic | 9.78 | -84.61 | Siles & Baker (2020) |
| *Xenoctenes* | *hirsuta* | Hi A | **ICN 22829 *** | MK936332 | MK936364 | Col | 3.88 | -76.43 | This study |
| *Xenoctenes* | *hirsuta* | Hi A | **ICN 19413 *** | MK936345 |  | Col | 11.35 | -72.14 | This study |
| *Xenoctenes* | *hirsuta* | Hi A | **ICN Temp D3M 541 *** | MK936350 |  | Col | 6.61 | -74.08 | This study |
| *Xenoctenes* | *hirsuta* | Hi B | **ICN 24463 *** | MK936344 | MK936363 | Col | 3.81 | -73.86 | This study |
| *Xenoctenes* | *hirsuta* | Hi B | **ICN Temp ATG161 *** | MK936352 |  | Col | -1.33 | -69.56 | This study |
| *Xenoctenes* | *hirsuta* | Hi B | **ICN 23867 *** | MK936351 | MK936362 | Col | 2.53 | -72.81 | This study |
