# Supplementary figures and images for: Beginning the quest: phylogenetic hypothesis and identification of evolutionary lineages in bats of the genus Micronycteris (Chiroptera, Phyllostomidae)
Source: Zookeys. 2021 Apr 6;1028:135–59. doi: 10.3897/zookeys.1028.60955 (PMC8044067; doi:10.3897/zookeys.1028.60955)

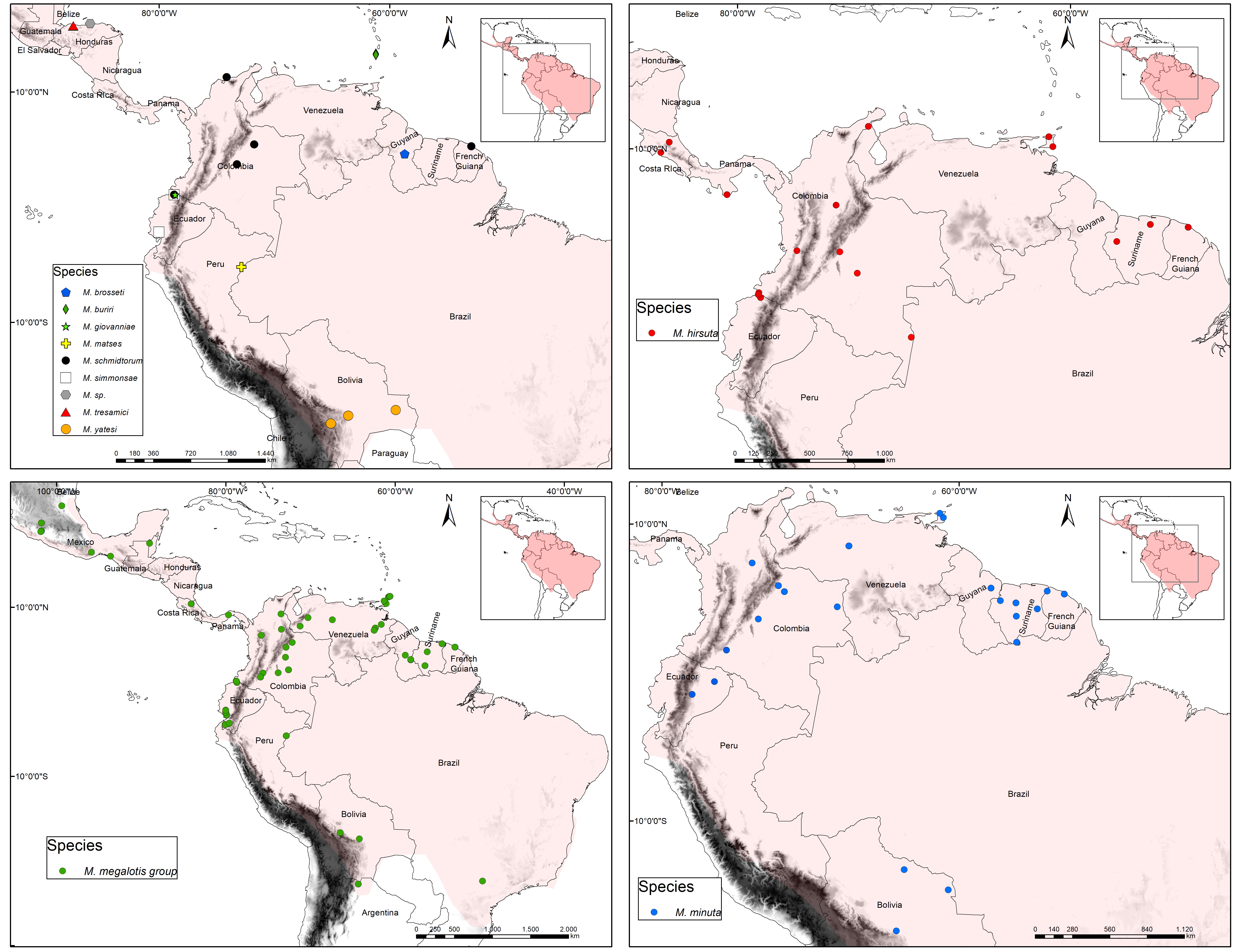

Supplement: Supplementary material 2 — Figure S1. Geographic location of sequences used in molecular analyses [file zookeys-1028-135-s002.jpg]
