# Supplementary material for: Beginning the quest: phylogenetic hypothesis and identification of evolutionary lineages in bats of the genus Micronycteris (Chiroptera, Phyllostomidae)
Source: Zookeys. 2021 Apr 6;1028:135–59. doi: 10.3897/zookeys.1028.60955 (PMC8044067; doi:10.3897/zookeys.1028.60955)
Supplement: Supplementary material 3 — Tables S2–S2.4 [file zookeys-1028-135-s003.docx]

Table S2. Average *p*-genetic distances within (diagonal) and between (below the diagonal) species and clades per subgenus of *Micronycteris* based on the *Cytb* gene. Acronyms represent the lineages of *M. megalotis*, *M. hirsuta* and *M. minuta* species complex.

Table S2.1. Subgenus *Micronycteris*.

|  | *Micronycteris* | 1 | 2 | 3 | 4 | 5 | 6 | 7 | 8 | 9 | 10 | 11 | 12 | 13 |
| --- | --- | --- | --- | --- | --- | --- | --- | --- | --- | --- | --- | --- | --- | --- |
| 1 | *M. buriri* | **0.00** |  |  |  |  |  |  |  |  |  |  |  |  |
| 2 | *M. giovanniae* | 7.15 | **NA** |  |  |  |  |  |  |  |  |  |  |  |
| 3 | *M. matses* | 4.63 | 5.32 | **0.00** |  |  |  |  |  |  |  |  |  |  |
| 4 | *M.* sp. | 6.84 | 6.61 | 5.96 | **NA** |  |  |  |  |  |  |  |  |  |
| 5 | Me A | 5.22 | 5.87 | 2.90 | 5.85 | **2.00** |  |  |  |  |  |  |  |  |
| 6 | Me B | 5.30 | 7.18 | 5.39 | 7.61 | 5.29 | **NA** |  |  |  |  |  |  |  |
| 7 | Me C | 6.33 | 7.26 | 5.53 | 7.69 | 5.32 | 5.24 | **1.00** |  |  |  |  |  |  |
| 8 | Me D | 5.45 | 6.02 | 4.85 | 7.57 | 4.82 | 4.68 | 4.40 | **2.00** |  |  |  |  |  |
| 9 | Me E | 4.94 | 6.18 | 5.10 | 7.33 | 4.67 | 5.75 | 5.96 | 4.87 | **NA** |  |  |  |  |
| 10 | Me F | 4.50 | 6.32 | 4.47 | 6.41 | 4.53 | 5.27 | 5.93 | 5.14 | 4.02 | **2.00** |  |  |  |
| 11 | Me G | 4.24 | 6.93 | 4.63 | 6.93 | 4.87 | 5.68 | 6.11 | 5.58 | 5.14 | 4.91 | **2.00** |  |  |
| 12 | Me H | 2.76 | 6.61 | 4.19 | 6.29 | 4.58 | 5.64 | 5.71 | 5.80 | 4.66 | 4.57 | 3.80 | **0.01** |  |
| 13 | Me I | 1.90 | 6.68 | 4.89 | 7.06 | 5.35 | 5.82 | 6.18 | 5.63 | 4.96 | 4.77 | 4.18 | 2.79 | **0.00** |

Table S2.2. Subgenus *Xenonectes*.

|  | *Xenonectes* | 1 | 2 |
| --- | --- | --- | --- |
| 1 | Hi A | **2.00** |  |
| 2 | Hi B | 8.57 | **2.00** |

Table S2.3. Subgenus *Leuconycteris*.

|  | *Leuconycteris* | 1 | 2 |
| --- | --- | --- | --- |
| 1 | *M. brosseti* | **0.00** |  |
| 2 | *M. schmidtorum* | 9.34 | **2.00** |

Table S2.4. Subgenus *Schizonycteris*.

|  | *Schizonycteris* | 1 | 2 | 3 | 4 | 5 | 6 | 7 | 8 | 9 | 10 |
| --- | --- | --- | --- | --- | --- | --- | --- | --- | --- | --- | --- |
| 1 | *M. tresamici* | **2.00** |  |  |  |  |  |  |  |  |  |
| 2 | *M. simmonsae* | 10.58 | **2.00** |  |  |  |  |  |  |  |  |
| 3 | *M. yatesi* | 10.70 | 7.98 | **1.00** |  |  |  |  |  |  |  |
| 4 | Mi A | 10.88 | 6.98 | 8.73 | **1.00** |  |  |  |  |  |  |
| 5 | Mi B | 10.44 | 7.32 | 7.94 | 4.34 | **1.00** |  |  |  |  |  |
| 6 | Mi C | 10.37 | 7.22 | 8.03 | 4.36 | 3.42 | **1.00** |  |  |  |  |
| 7 | Mi D | 9.81 | 7.59 | 8.08 | 6.09 | 4.95 | 5.43 | **0.00** |  |  |  |
| 8 | Mi E | 10.56 | 8.65 | 9.58 | 4.95 | 5.77 | 5.36 | 6.59 | **0.00** |  |  |
| 9 | Mi F | 10.69 | 7.65 | 8.72 | 5.30 | 5.09 | 5.04 | 5.23 | 5.19 | **0.00** |  |
| 10 | Mi G | 9.88 | 7.04 | 7.69 | 3.92 | 3.69 | 4.00 | 4.40 | 4.14 | 3.50 | **1.00** |
