# Supplementary material for: Beginning the quest: phylogenetic hypothesis and identification of evolutionary lineages in bats of the genus Micronycteris (Chiroptera, Phyllostomidae)
Source: Zookeys. 2021 Apr 6;1028:135–59. doi: 10.3897/zookeys.1028.60955 (PMC8044067; doi:10.3897/zookeys.1028.60955)

Supplementary information S5. Results of the delimitation analyses:

**BPP Analyses**

The bPTP delimitation search was performed using 500 000 Markov chain Monte Carlo (MCMC) generations, with thinning set to 100 and a burn-in of 25% initial samples. Bold indicate posterior probabilities >95%.

# Most supported partition found by simple heuristic search

Species 1 (support = 0.934)

brosseti_KU155163, brosseti_KU155162

Species 2 (support = **1.000**)

giovanniae_QCAZ7200

Species 3 (support = **1.000**)

sp_TTU104168

Species 4 (support = 0.898)

minuta_MHNCM175, minuta_MHNCM186, minuta_MSB235221

Species 5 (support = **1.000**)

megalotis_complex_ICN23839

Species 6 (support = **1.000**)

megalotis_complex_ROM111099

Species 7 (support = 0.939)

matses_AMNH272814, matses_AMNH273095, matses_AMNH273044

Species 8 (support = **0.957**)

minuta_AMNH267874, minuta_AMNH267875, minuta_CM76769

Species 9 (support = 0.894)

minuta_USNM582262, minuta_USNM582263, minuta_CM63584, minuta_CM68639

Species 10 (support = 0.666)

megalotis_complex_ICN22674, megalotis_complex_ICN21568

Species 11 (support = 0.878)

megalotis_complex_ICN24495, megalotis_complex_ICN24494

Species 12 (support = 0.834)

megalotis_complex_ICN23731, megalotis_complex_ICN23730

Species 13 (support = 0.880)

hirsuta_CM97178, hirsuta_CM68638, hirsuta_AMNH267858, hirsuta_AMNH267857, hirsuta_AMNH267860, hirsuta_CM97176, hirsuta_CM68387

Species 14 (support = **0.999**)

simmonsae_TTU103253

Species 15 (support = **0.999**)

simmonsae_TTU103198

Species 16 (support = **0.969**)

megalotis_complex_CM63575, megalotis_complex_CM63577

Species 17 (support = 0.864)

megalotis_complex_AMNH267864, megalotis_complex_AMNH267090, megalotis_complex_AMNH267862

Species 18 (support = 0.840)

megalotis_complex_TK167804, megalotis_complex_TK167801

Species 19 (support = 0.811)

megalotis_complex_TTU103311, megalotis_complex_TTU103284, megalotis_complex_TTU103436, megalotis_complex_TTU103387, megalotis_complex_TTU103491, megalotis_complex_TTU85289, megalotis_complex_TTU102918, megalotis_complex_TTU85389, megalotis_complex_TTU103800, megalotis_complex_TTU102398, megalotis_complex_TTU102448, megalotis_complex_TTU102602

Species 20 (support = **0.998**)

schmidtorum_AMNH267854

Species 21 (support = 0.881)

hirsuta_TTU85449, hirsuta_TTU103117, hirsuta_TTU85428

Species 22 (support = **0.999**)

tresamici_TTU103880

Species 23 (support = **0.999**)

tresamici_TTU61057

Species 24 (support = 0.788)

minuta_ICNTGEP931, minuta_ICNTFSC171, minuta_ICN22939, minuta_ICNTDRG086, minuta_TTU33282, minuta_TTU33280, minuta_TTU33278, minuta_CM97184, minuta_TTU43946

Species 25 (support = **0.990**)

minuta_ICNIUSTJEC193

Species 26 (support = **0.997**)

megalotis_complex_CM68389

Species 27 (support = 0.592)

megalotis_complex_CM78299, megalotis_complex_CM78295, megalotis_complex_CM78294, megalotis_complex_CM78297, megalotis_complex_ROM108745, megalotis_complex_CM97180, megalotis_complex_CM76768, megalotis_complex_AMNH267865, megalotis_complex_AMNH267866, megalotis_complex_AMNH267868, megalotis_complex_AMNH267867, megalotis_complex_AMNH267097

Species 28 (support = 0.692)

hirsuta_ICNATG161, hirsuta_ICN23867

Species 29 (support = **0.999**)

hirsuta_ICN24463

Species 30 (support = 0.861)

hirsuta_ICNTD3M541

Species 31 (support = 0.861)

hirsuta_ICN19413

Species 32 (support = 0.881)

megalotis_complex_UNSMZM29489, megalotis_complex_TTU43944, megalotis_complex_UNSMZM29493, megalotis_complex_UNSMZM29476

Species 33 (support = 0.921)

hirsuta_ICN22829

Species 34 (support = 0.921)

hirsuta_MSB94371

Species 35 (support = 0.922)

hirsuta_TK167825

Species 36 (support = 0.922)

hirsuta_TK167812

Species 37 (support = 0.559)

megalotis_complex_ICN24484, megalotis_complex_ROM104195, megalotis_complex_ICN21124, megalotis_complex_ICN17736, megalotis_complex_CM97182, megalotis_complex_ICN21000, megalotis_complex_TTU33276, megalotis_complex_ICN22485, megalotis_complex_ICN23203, megalotis_complex_ICNTDRG076, megalotis_complex_CM78291, megalotis_complex_CM78292

Species 38 (support = **0.967**)

megalotis_complex_CM78298

Species 39 (support = 0.583)

schmidtorum_ICN22940, schmidtorum_ICN21576

Species 40 (support = 0.660)

megalotis_complex_AMNH273081, megalotis_complex_AMNH273098, megalotis_complex_AMNH273169, megalotis_complex_AMNH273072, megalotis_complex_MUSM13210

Species 41 (support = 0.712)

megalotis_complex_CML7552, megalotis_complex_CML7553, megalotis_complex_MHNCM189, megalotis_complex_MHNCM144

Species 42 (support = 0.569)

buriri_TTU105972

Species 43 (support = 0.551)

buriri_TTU105641, buriri_TTU105352, buriri_TTU105535, buriri_TTU105642

Species 44 (support = 0.550)

minuta_ICN23912

Species 45 (support = 0.550)

minuta_ICN24472

Species 46 (support = 0.488)

schmidtorum_TTU103196

Species 47 (support = 0.488)

schmidtorum_ICN24479

Species 48 (support = 0.776)

megalotis_complex_TTU36534, megalotis_complex_AMNH274585, megalotis_complex_TTU82624, megalotis_complex_TTU35355

Species 49 (support = 0.910)

megalotis_complex_TK45332, megalotis_complex_TK45354, megalotis_complex_TK45487, megalotis_complex_TK45489, megalotis_complex_TK45330

Species 50 (support = 0.597)

minuta_ICN24465

Species 51 (support = 0.522)

minuta_ROM104067, minuta_TTU84825

Species 52 (support = 0.812)

minuta_CM77112, minuta_CM77111, minuta_TTU109879

Species 53 (support = 0.867)

minuta_TTU106007

Species 54 (support = 0.840)

yatesi_MHNCM157, yatesi_MHNCM141

Species 55 (support = 0.884)

yatesi_CBF6154

Figure S4.1. bPTP likelihood trace plot convergence of bPTP analysis.


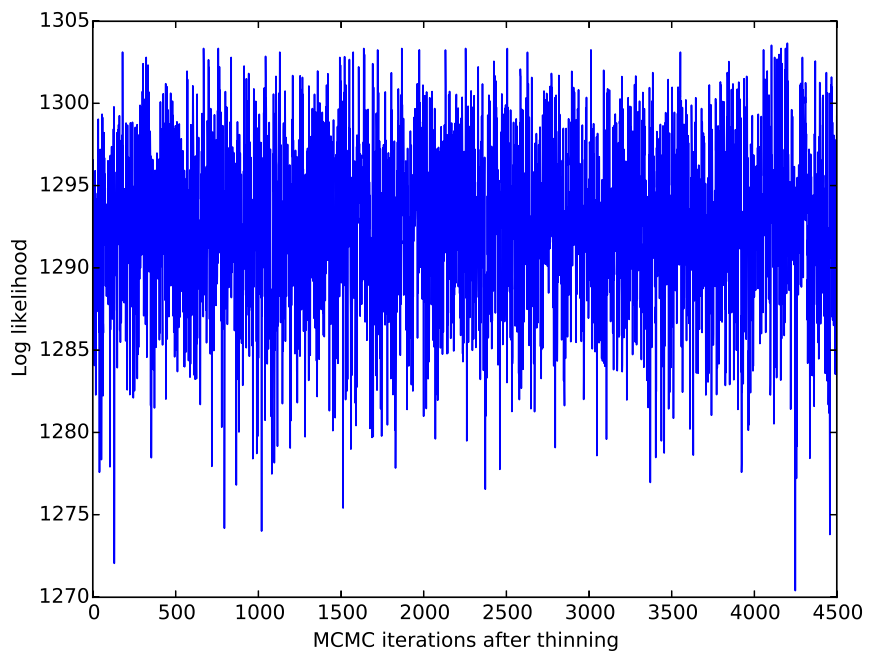


Figure S4.2. bPTP tree with delimitation results in red.


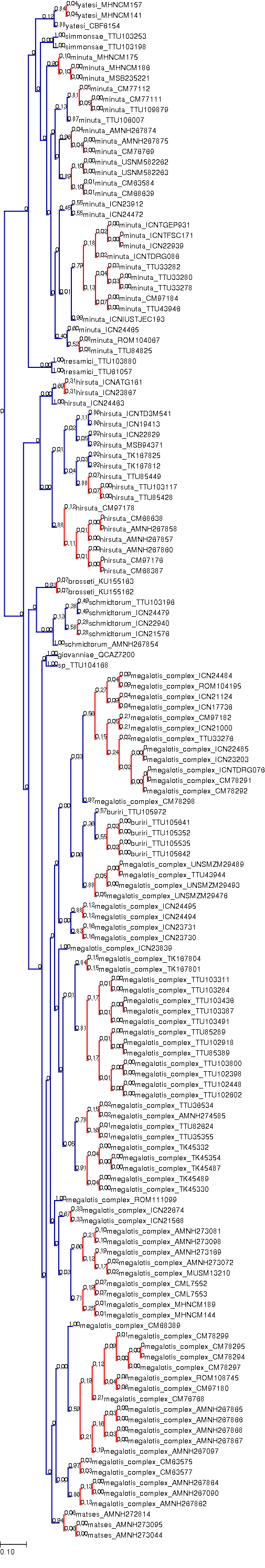


**GMYC Analyses**

GMYC single-threshold results

Method: single

likelihood of null model: -1414.993

maximum likelihood of GMYC model: 1105.419

likelihood ratio: 5040.823

result of LR test: 0***

number of ML clusters: 35

confidence interval: 1-39

number of ML entities: 48

confidence interval: 2-146

threshold time: -0.008590693

Figure 3. Single-threshold models indicating the threshold time between Yule and coalescent process as estimated by the likelihood implementation of the general mixed Yule coalescent model (GMYC).


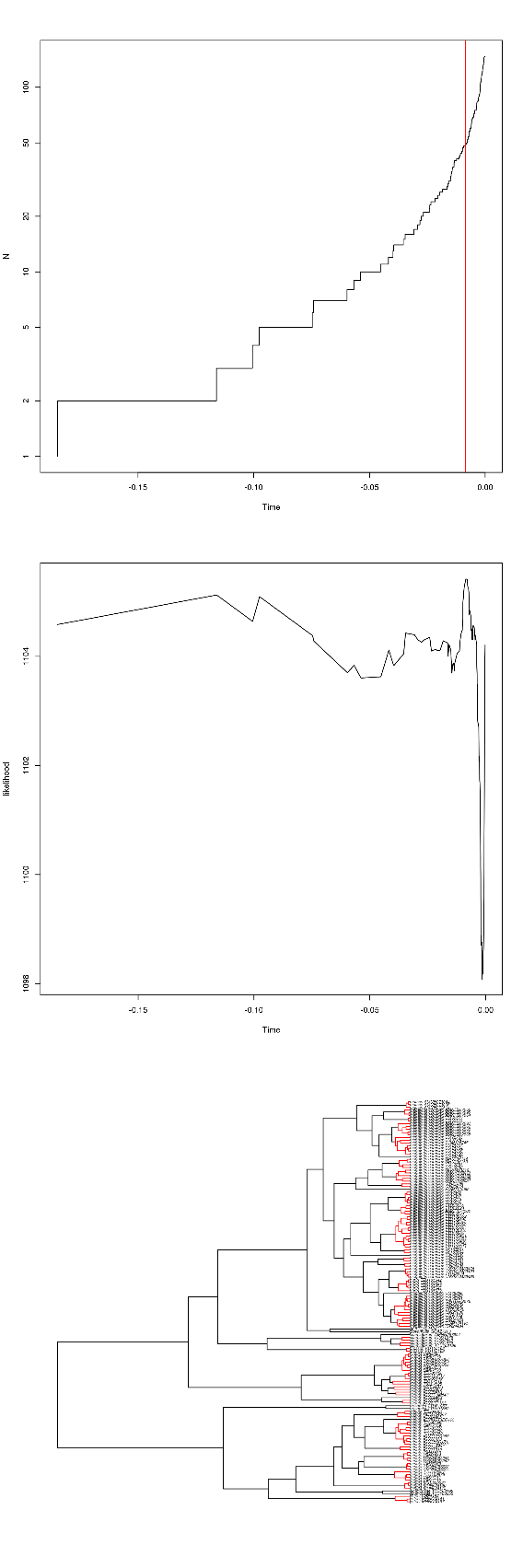


Figure S4.3. GYMC tree with delimitation results in red.


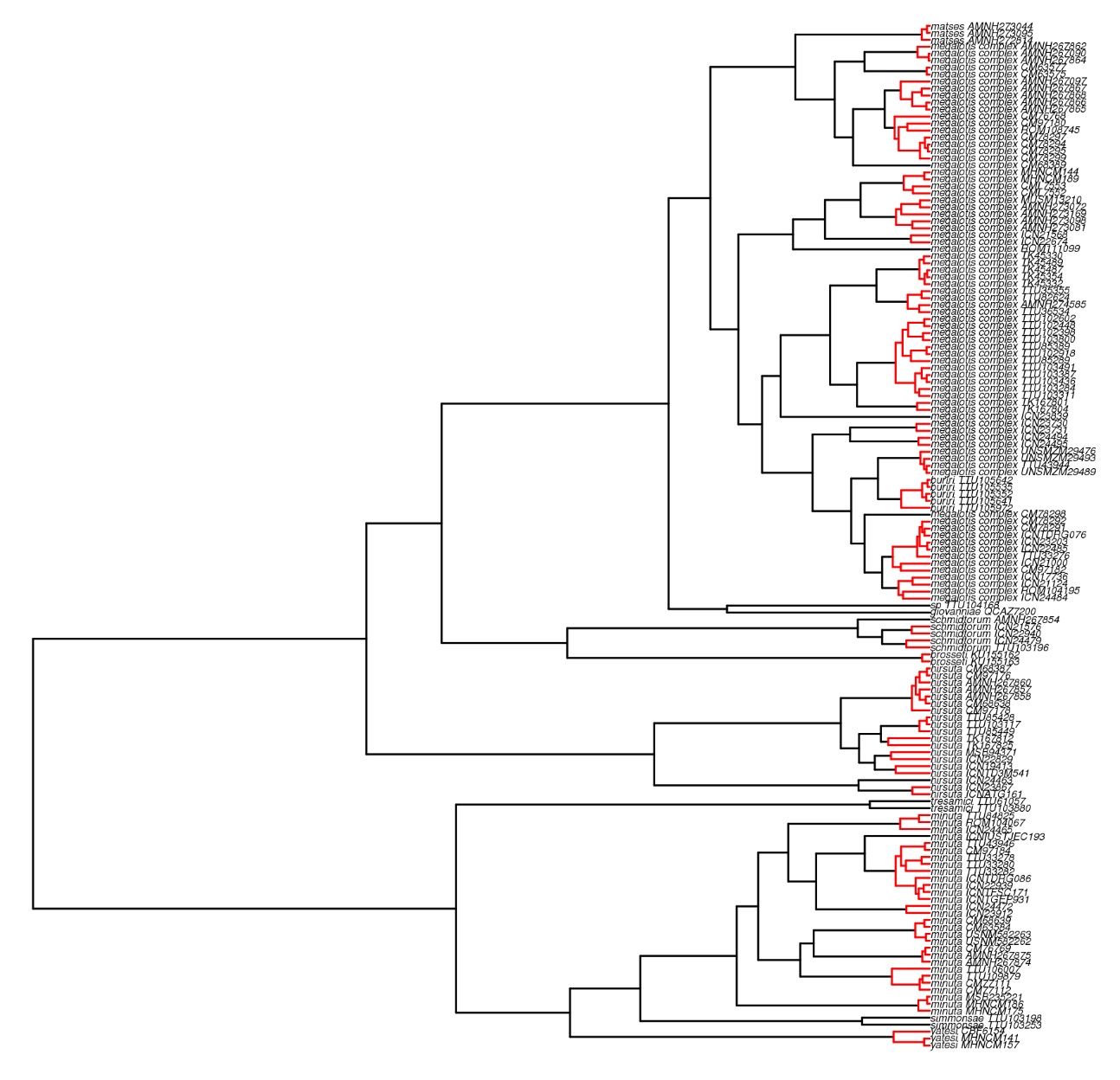


**MPTP Analyses**

Delimitation results: Number of delimited species: 33

Species 1:

yatesi_MHNCM157

yatesi_MHNCM141

yatesi_CBF6154

Species 2:

simmonsae_TTU103253

simmonsae_TTU103198

Species 3:

minuta_MHNCM175

minuta_MHNCM186

minuta_MSB235221

Species 4:

minuta_CM77112

minuta_CM77111

minuta_TTU109879

minuta_TTU106007

Species 5:

minuta_AMNH267874

minuta_AMNH267875

minuta_CM76769

Species 6:

minuta_USNM582262

minuta_USNM582263

minuta_CM63584

minuta_CM68639

Species 7:

minuta_ICN23912

minuta_ICN24472

Species 8:

minuta_ICNTGEP931

minuta_ICNTFSC171

minuta_ICN22939

minuta_ICNTDRG086

minuta_TTU33282

minuta_TTU33280

minuta_TTU33278

minuta_CM97184

minuta_TTU43946

Species 9:

minuta_ICNIUSTJEC193

Species 10:

minuta_ICN24465

minuta_ROM104067

minuta_TTU84825

Species 11:

tresamici_TTU103880

tresamici_TTU61057

Species 12:

hirsuta_ICNATG161

hirsuta_ICN23867

hirsuta_ICN24463

Species 13:

hirsuta_ICNTD3M541

hirsuta_ICN19413

hirsuta_ICN22829

hirsuta_MSB94371

hirsuta_TK167825

hirsuta_TK167812

hirsuta_TTU85449

hirsuta_TTU103117

hirsuta_TTU85428

Species 14:

hirsuta_CM97178

hirsuta_CM68638

hirsuta_AMNH267858

hirsuta_AMNH267857

hirsuta_AMNH267860

hirsuta_CM97176

hirsuta_CM68387

Species 15:

brosseti_KU155163

brosseti_KU155162

Species 16:

schmidtorum_TTU103196

schmidtorum_ICN24479

schmidtorum_ICN22940

schmidtorum_ICN21576

schmidtorum_AMNH267854

Species 17:

giovanniae_QCAZ7200

sp_TTU104168

Species 18:

megalotis_complex_ICN24484

megalotis_complex_ROM104195

megalotis_complex_ICN21124

megalotis_complex_ICN17736

megalotis_complex_CM97182

megalotis_complex_ICN21000

megalotis_complex_TTU33276

megalotis_complex_ICN22485

megalotis_complex_ICN23203

megalotis_complex_ICNTDRG076

megalotis_complex_CM78291

megalotis_complex_CM78292

megalotis_complex_CM78298

buriri_TTU105972

buriri_TTU105641

buriri_TTU105352

buriri_TTU105535

buriri_TTU105642

megalotis_complex_UNSMZM29489

megalotis_complex_TTU43944

megalotis_complex_UNSMZM29493

megalotis_complex_UNSMZM29476

Species 19:

megalotis_complex_ICN24495

megalotis_complex_ICN24494

Species 20:

megalotis_complex_ICN23731

megalotis_complex_ICN23730

Species 21:

megalotis_complex_ICN23839

Species 22:

megalotis_complex_TK167804

megalotis_complex_TK167801

Species 23:

megalotis_complex_TTU103311

megalotis_complex_TTU103284

megalotis_complex_TTU103436

megalotis_complex_TTU103387

megalotis_complex_TTU103491

megalotis_complex_TTU85289

megalotis_complex_TTU102918

megalotis_complex_TTU85389

megalotis_complex_TTU103800

megalotis_complex_TTU102398

megalotis_complex_TTU102448

megalotis_complex_TTU102602

Species 24:

megalotis_complex_TTU36534

megalotis_complex_AMNH274585

megalotis_complex_TTU82624

megalotis_complex_TTU35355

Species 25:

megalotis_complex_TK45332

megalotis_complex_TK45354

megalotis_complex_TK45487

megalotis_complex_TK45489

megalotis_complex_TK45330

Species 26:

megalotis_complex_ROM111099

Species 27:

megalotis_complex_ICN22674

megalotis_complex_ICN21568

Species 28:

megalotis_complex_AMNH273081

megalotis_complex_AMNH273098

megalotis_complex_AMNH273169

megalotis_complex_AMNH273072

megalotis_complex_MUSM13210

megalotis_complex_CML7552

megalotis_complex_CML7553

megalotis_complex_MHNCM189

megalotis_complex_MHNCM144

Species 29:

megalotis_complex_CM68389

Species 30:

megalotis_complex_CM78299

megalotis_complex_CM78295

megalotis_complex_CM78294

megalotis_complex_CM78297

megalotis_complex_ROM108745

megalotis_complex_CM97180

megalotis_complex_CM76768

megalotis_complex_AMNH267865

megalotis_complex_AMNH267866

megalotis_complex_AMNH267868

megalotis_complex_AMNH267867

megalotis_complex_AMNH267097

Species 31:

megalotis_complex_CM63575

megalotis_complex_CM63577

Species 32:

megalotis_complex_AMNH267864

megalotis_complex_AMNH267090

megalotis_complex_AMNH267862

Species 33:

matses_AMNH272814

matses_AMNH273095

matses_AMNH273044

Figure S4.4. Species delimitation results according to mPTP model in red.


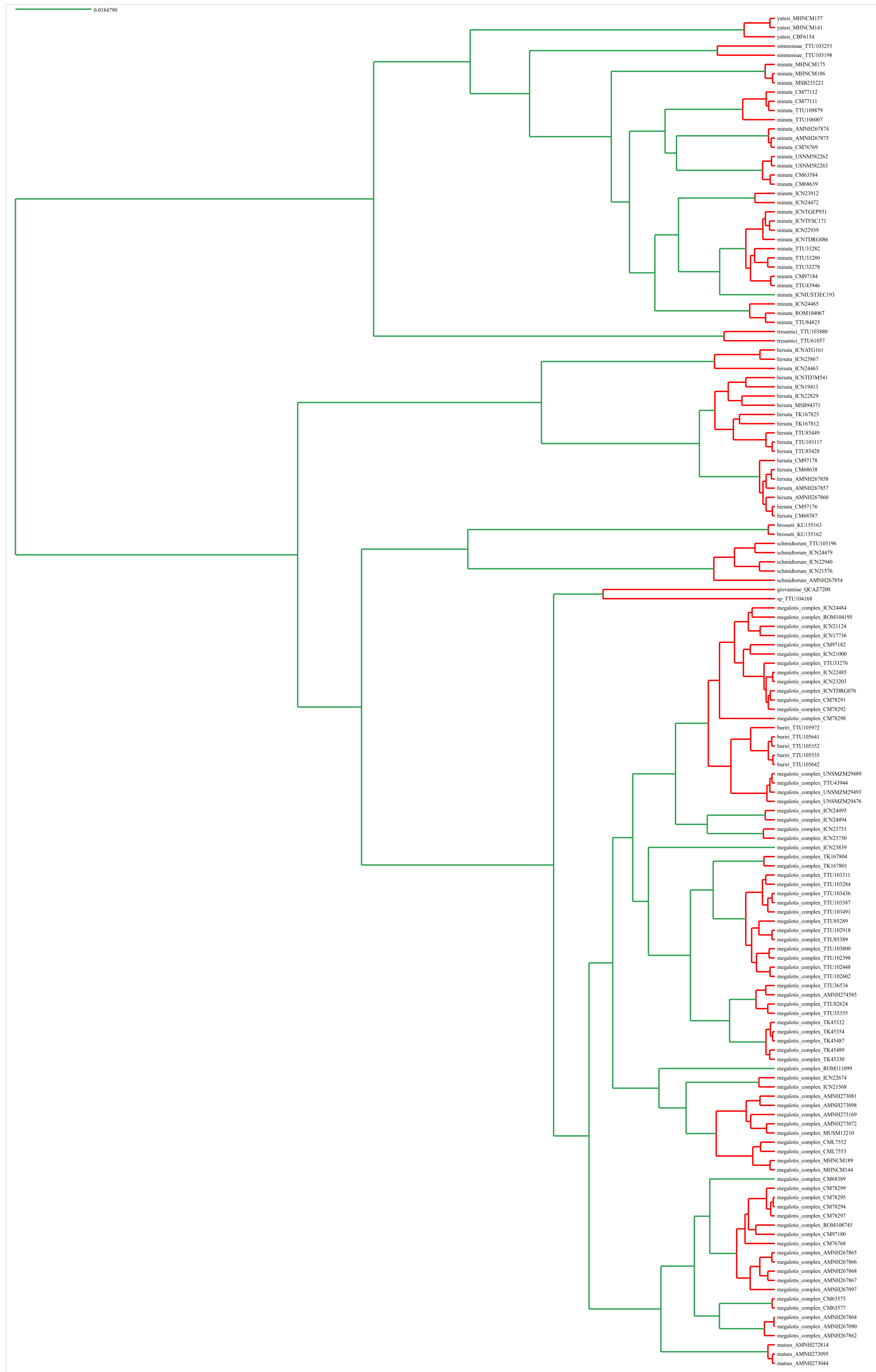

Supplement: Supplementary material 4 — Results of the delimitation analyses [file zookeys-1028-135-s004.docx]
